# Exploiting collateral sensitivity in the evolution of resistance to tyrosine kinase inhibitors in soft tissue sarcomas

**DOI:** 10.1038/s42003-025-08652-1

**Published:** 2025-08-08

**Authors:** Mark L. Elms, Andrew D. Jenks, Avirup Chowdhury, Lukas Krasny, Madhumeeta Chadha, Kaan Low, Peter T. Harrison, William G. Kerrison, Robin L. Jones, Paul H. Huang

**Affiliations:** 1 Division of Cancer Biology, The Institute of Cancer Research, London, UK; 2 The Royal Marsden NHS Foundation Trust, London, UK; 3 Division of Clinical Studies, The Institute of Cancer Research, London, UK

**Keywords:** Sarcoma, Targeted therapies

## Abstract

Broad-spectrum multi-target tyrosine kinase inhibitors (mTKIs) are clinically approved for the treatment of soft tissue sarcomas (STS). However, acquired resistance inevitably arises in the majority of STS patients. There is therefore an urgent need to identify new strategies to overcome resistance and achieve durable treatment responses. Here we show that STS cells that acquire resistance to clinically relevant mTKIs are cross-resistant to one another and sequential treatment does not delay the acquisition of drug resistance. Instead, we find that en route to acquiring drug resistance, STS cells develop collateral sensitivities to alternative drugs. We demonstrate that the mTKI sitravatinib rapidly induces collateral sensitivity to the FGFR inhibitor infigratinib which can be exploited for adaptive therapy to suppress STS cell growth. This study provides proof-of-principle that collateral sensitivity may be an effective strategy for overcoming resistance to mTKIs and this novel approach should be explored in the design of future trials.

## Abstract (web summary)

Sarcoma treatments are limited due to resistance. Analysis of clinically relevant tyrosine kinase inhibitors demonstrates that sarcoma cells develop collateral sensitivities to alternative drugs which can be exploited for adaptive therapies.

## Introduction

Broad-spectrum multi-target tyrosine kinase inhibitors (mTKIs) have been approved for use in a number of cancer types including renal cell carcinoma, hepatocellular carcinoma, colorectal cancer and soft tissue sarcomas (STS)^1–4^. Although widely thought to be anti-angiogenic agents, their inherent polypharmacological properties, as revealed by in vitro kinase assay screens and chemical proteomics^5–7^, means that the exact mechanisms of action for these drugs are poorly understood. In cancers driven by mutant oncogenes, such as *KIT* or *PDGFRA* mutant gastrointestinal stromal tumours^8,9^ and *EGFR* or *EML4-ALK* mutant lung cancers^10–12^, sequential treatments with up to fourth-line kinase inhibitor therapy have achieved long-term disease control and improved patient outcomes. This sequential approach has been less successful with mTKIs in renal cell carcinoma and STS^13–16^. New strategies are therefore needed to optimise the use of mTKIs in these diseases.

Since current clinical management in advanced disease focuses on treating drug resistant tumours at relapse, one promising option is to define new vulnerabilities that arise when tumour cells evolve to acquire secondary drug resistance – a phenomenon known as collateral sensitivity^17,18^. By exploiting collateral sensitivities, it is possible to use an “evolutionary steering” strategy to control drug resistance^19^. This approach has been extensively studied in tackling antibiotic resistance in bacterial infections^17,20,21^. In the context of cancer, studies evaluating kinase inhibitor collateral sensitivities have to date been restricted to drugs that block the oncogenes *BCR-ABL*, *EML4-ALK* and mutant *EGFR*^19,22,23^. Since mTKIs can simultaneously inhibit multiple oncogenic pathways and target distinct subclones within a heterogeneous tumour population^24^, it is currently not known if their broad-spectrum properties impact the evolutionary “trade-offs” that lead to collateral sensitivity.

In this study, we sought to address two key questions: 1. Is therapy with different mTKIs effective in overcoming or delaying acquired resistance when used in sequence? 2. Does treatment with mTKIs lead to the development of collateral sensitivities that could be exploited for evolutionary steering and adaptive therapy? Employing STS cell line models and four different mTKIs, we show that while acquisition of resistance to any one mTKI confers cross-resistance to the other mTKIs under study, resistance to some mTKIs confers collateral sensitivities to a spectrum of alternative drugs. These findings have the potential to inform the design of future clinical trials evaluating mTKI adaptive therapy in STS and beyond.

## Results

### A subset of sarcoma cell lines are sensitive to multiple mTKIs

**Fig. 1:**
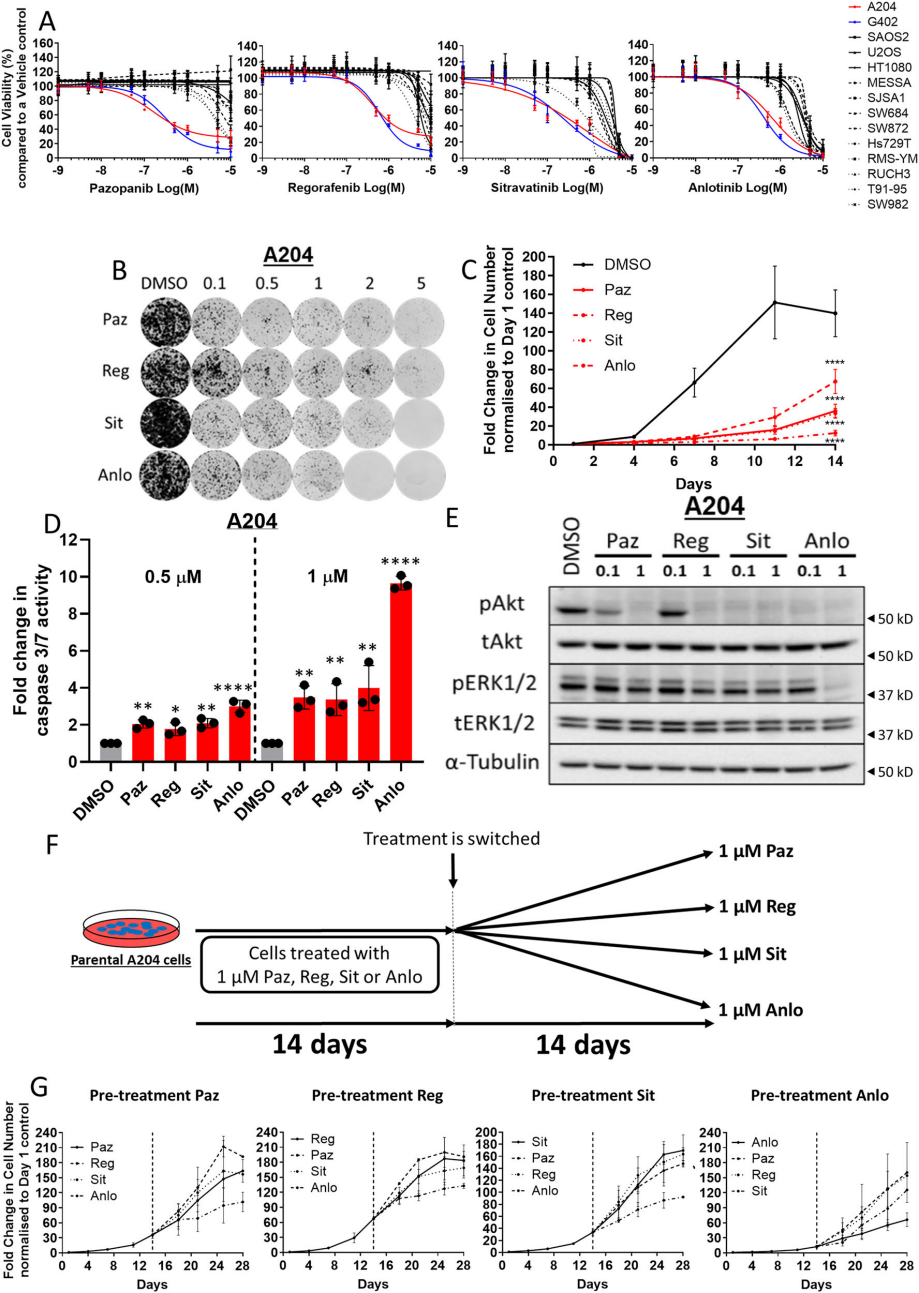
Malignant rhabdoid tumour cells are sensitive to pazopanib, regorafenib, sitravatinib and anlotinib. **A** Dose response assays of a panel of 14 sarcoma cell lines treated for 72 h with increasing concentrations of mTKI to determine IC_50_ values (Table S1). Cell viability was normalised to DMSO control. Error bars represent standard deviation (*n* = 3). **B** Colony formation assays of A204 cells treated with increasing concentrations of mTKIs over a period of 2 weeks. Image is representative of three separate experiments. **C** Growth curve assays of A204 to measure the fold change in cell number over a period of 2 weeks. Fold change was normalised to day 1 control (*n* = 4). Error bars represent standard deviation. Statistical analysis was undertaken using one-way ANOVAs with Dunnett’s multiple comparison tests (DMSO control). **D** Bar plots displaying the fold change in caspase 3/7 activity in the A204 cells treated with two concentrations of mTKIs for 24 h. Fold change was normalised to DMSO control (*n* = 3). Error bars represent standard deviation. Statistical analysis was undertaken using one-way ANOVA with Dunnett’s multiple comparison tests (DMSO control) (* = *p* ≤ 0.05, ** = *p* ≤ 0.01, **** = *p* ≤ 0.0001). **E** Immunoblot of Akt and ERK1/2 signalling modulation in A204 cells after 6 h of treatment with either 0.1 or 1 μM of mTKIs. Image is representative of two separate experiments. (**F**) Schematic of experimental design for temporal experiment. A204 cells were pre-treated with mTKIs for 14 days and then switched to an alternative mTKI for a further 14 days. **G** Growth curve assays of A204 cells to measure the fold change in cell number over a period of 4 weeks. Fold change was normalised to day 1 control (*n* = 2). Error bars represent standard deviation of the mean. Paz Pazopanib, Reg Regorafenib, Sit Sitravatinib and Anlo Anlotinib.

Pazopanib is a mTKI that has been approved for use in non-adipocytic STS^3,25^. In addition to pazopanib, three other mTKIs (regorafenib, sitravatinib and anlotinib) are currently undergoing or have completed evaluation in STS clinical trials^26–30^. These mTKIs each target a spectrum of tyrosine kinases with both shared and exclusive kinase targets^5,28,31^. Subjecting a panel of 14 sarcoma cell lines to mTKI treatment finds that only the malignant rhabdoid tumour cell lines A204 and G402 were sensitive to all four inhibitors (Fig. 1A, Table S1). This result was independently confirmed in colony formation (Fig. 1B and Fig. S1A–C), cell growth (Fig. 1C and Figure S1D) and apoptosis (Fig. 1D and Figure S1E) assays. Across the four drugs, anlotinib was the most potent mTKI displaying the strongest reduction in cell growth (Fig. 1C) and increase in apoptosis (Fig. 1D) in the A204 cells. While in the G402 cell line, both sitravatinib and anlotinib had comparatively more potent effects on cell growth (Figure S1D) and apoptosis (Figure S1E) than pazopanib and regorafenib. Consistent with our previously published observations with pazopanib^32^, all of the mTKIs under investigation had a dose dependent effect on the pAKT pathway in both cell lines (Fig. 1E and S1F). There was no reduction in the phosphorylation of Erk in both cell lines for all drug doses, with the exception of anlotinib at 1 µM dose in the A204 cells (Fig. 1E and Figure S1F). Despite the ability of these drugs to reduce cell growth, as expected, this effect was transient and cells ultimately regained the ability to grow in the presence of drug (Figure S1G, H) in both cell lines, indicating that the cells would eventually acquire mTKI resistance.

To evaluate if cancer cell proliferation would be halted by the use of these mTKIs in sequence, we pre-treated the A204 parental cells with each of the 4 mTKIs for 14 days, following which the drug was either switched to one of the 3 remaining mTKIs or continued with the initial drug (Fig. 1F). Cell growth was then monitored for a further 14 days. Following pre-treatment with pazopanib, switching to regorafenib or sitravatinib displayed the same growth dynamics as continuous treatment with pazopanib (Fig. 1G). Only treatment with anlotinib reduced A204 cell growth (Fig. 1G) following pazopanib therapy, which is consistent with increased efficacy observed in first line treatment with anlotinib (Fig. 1C). Switching drugs after pre-treatment with regorafenib and sitravatinib had the same effect as pazopanib pre-treatment. In contrast, following pre-treatment with anlotinib, switching to pazopanib, regorafenib or sitravatinib led to an increase in cell growth compared to continued treatment with anlotinib, confirming the increased potency of anlotinib amongst the 4 mTKIs (Fig. 1G). The similar growth dynamics observed following drug switching with pazopanib, regorafenib or sitravatinib demonstrates that these three mTKIs would likely be ineffective in delaying acquired drug resistance when used in sequence.

### Cells that acquire resistance to distinct mTKIs converge on common molecular and phenotypic features

**Fig. 2:**
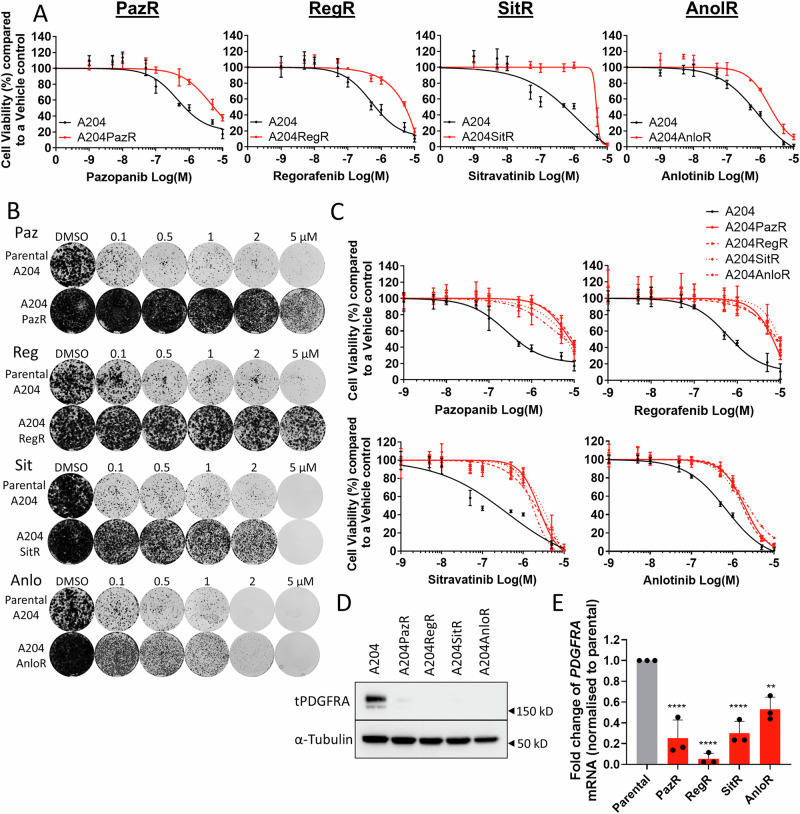
Characterisation of A204 mTKI-resistant sublines. **A** Dose response assays of A204PazR, A204RegR, A204SitR, and A204AnloR cells treated for 72 h with increasing concentrations of their respective inhibitor to determine IC_50_ values. Cell viability was normalised to DMSO control. Error bars represent standard deviation (*n* = 3). **B** Colony formation assays of A204, A204PazR, A204RegR, A204SitR and A204AnloR treated with increasing concentrations of their respective inhibitor over a period of 2 weeks. Image is representative of three separate experiments (*n* = 3). **C** Dose response assays of A204 mTKI-resistant sublines treated for 72 h with increasing concentrations of mTKIs to determine IC_50_ values. Cell viability was normalised to DMSO control. Error bars represent standard deviation (*n* = 3). **D** Immunoblot of baseline PDGFRA expression levels in A204 parental and TKI-resistant sublines. Image is representative of two separate experiments. **E** qPCR data displaying the fold change of *PDGFRA* mRNA, normalised to A204 parental cells. Error bars represent standard deviation (*n* = 3). Statistical analysis was undertaken using one-way ANOVAs with Dunnett’s multiple comparison tests (parental A204 control) (** = *p* ≤ 0.01, **** = *p* ≤ 0.0001). Paz Pazopanib, Reg Regorafenib, Sit Sitravatinib, Anlo Anlotinib.

One of the major challenges in the use of mTKIs is the acquisition of drug resistance. To study this process in more detail, we modelled acquired mTKI resistance in vitro by subjecting the A204 and G402 cells to long-term dose escalation treatment with each of the four mTKIs. In the A204 series, the resulting resistant sublines were named pazopanib (A204PazR), regorafenib (A204RegR), sitravatinib (A204SitR) and anlotinib (A204AnloR) resistant cells. Dose response (Fig. 2A and Figure S2A) and colony formation assays (Fig. 2B and Figure S2B) confirmed that these sublines had acquired drug resistance. Furthermore, we show that these four mTKIs were cross resistant to each other (Fig. 2C and Figure S2C). We previously found that the mechanism of pazopanib resistance in A204 cells was the result of a loss of PDGFRA expression, which was accompanied by a concomitant decrease in AKT phosphorylation^32^. In line with these results, we demonstrate that, as with A204PazR cells, A204RegR, A204SitR and A204AnloR sublines similarly lost PDGFRA expression at the protein and mRNA levels (Fig. 2D, E) and showed reduced baseline AKT phosphorylation (Figure S2D) compared to the parental A204 cell line, indicative of a shared mechanism of resistance. These experiments were repeated in the G402 cell line with similar results (Figure S3A–E), confirming the generalizability of our findings.

Given that switching of mTKIs when used in sequence was ineffective in delaying acquisition of drug resistance in the A204 parental cell line (Fig. 1G), we sought to determine if combinations of mTKIs were capable of sensitizing the panel of A204 mTKI resistant sublines. We find that combination treatment with any of the four mTKIs did not show any additive or synergistic effects (combination index <1) on cell viability (Table S2). These data provide additional evidence that the growth inhibition profile of these mTKIs are likely to be similar with no added benefit seen when combinations are used.

Collectively, these data indicate that despite being derived from long-term treatment with different mTKIs, the four acquired drug resistant sublines converge on multiple shared features including loss of PDGFRA expression, reduction in pAKT levels and cross resistance to each other.

### PDGFRA is the target of mTKIs in malignant rhabdoid tumour cells

**Fig. 3:**
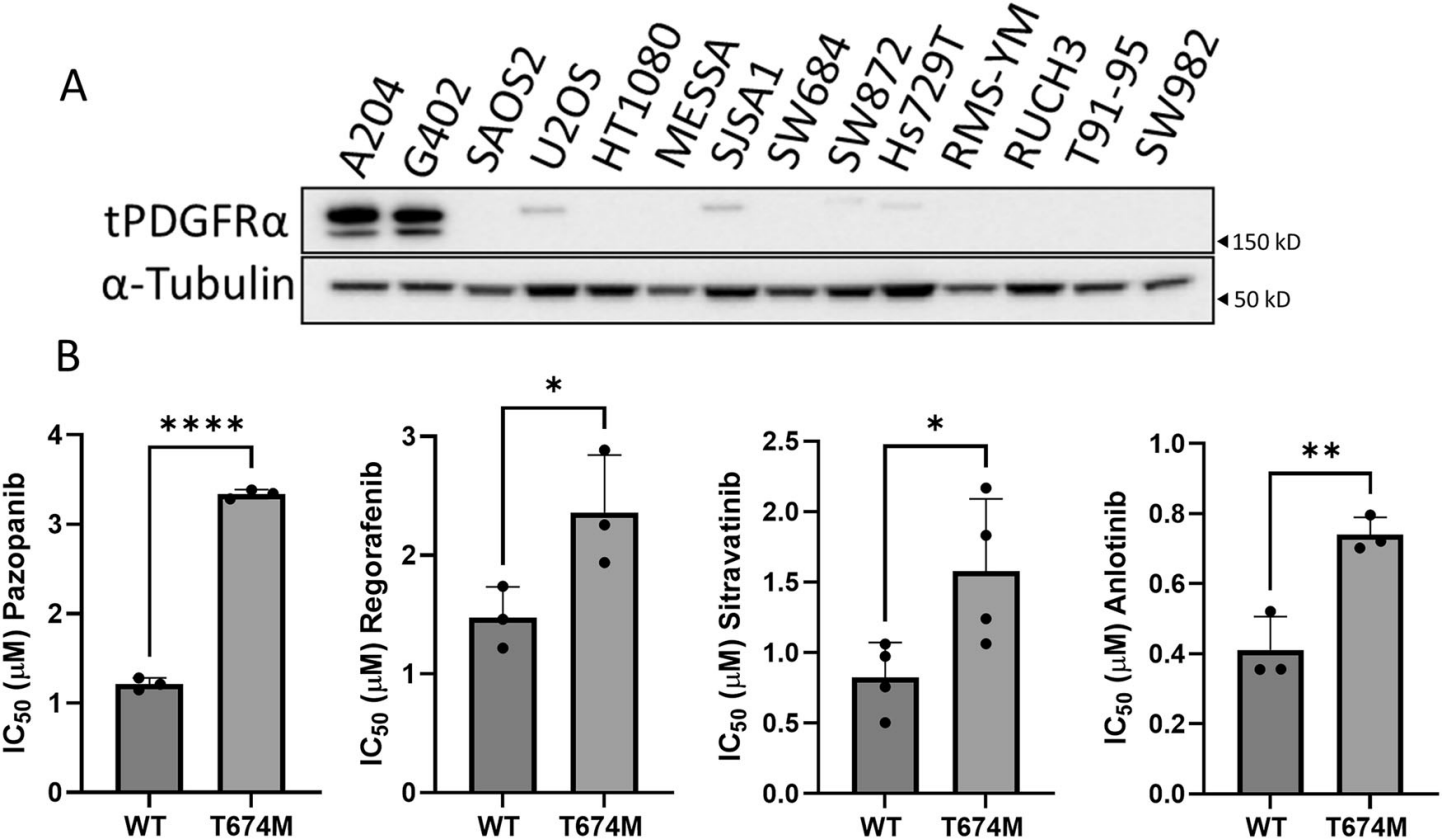
PDGFRA is the target of mTKIs in the malignant rhabdoid tumour cells. **A** Immunoblot of baseline PDGFRA expression levels in the sarcoma cell line panel. Image is representative of two separate experiments. **B** IC_50_ values of A204 cells expressing a wild type (WT) or gatekeeper mutant PDGFRA (T674M) protein, treated with respective inhibitors. Pazopanib, regorafenib, anolotinib (*n* = 3) and sitravatinib (*n* = 4). Error bars represent standard deviation. Statistical analysis was undertaken by Student’s unpaired T tests (* = *p* ≤ 0.05, ** = *p* ≤ 0.01, **** = *p* ≤ 0.0001).

Given that PDGFRA expression was diminished upon acquisition of resistance to the four mTKIs, we sought to determine the expression levels of this receptor across the panel of 12 sarcoma cell lines that harbour intrinsic resistance to the mTKIs. Consistent with the loss of PDGFRA expression in the acquired resistant sublines, all of the primary resistant sarcoma cell lines expressed low levels of PDGFRA compared to both parental A204 and G402 (Fig. 3A).

To investigate whether PDGFRA is the bona fide target of these four mTKIs in the malignant rhabdoid tumour cells, we utilised a gatekeeper mutant of PDGFRA (T674M) that is incapable of binding to the mTKIs^33^. If the different mTKIs harbour distinct modes of action that are independent of PDGFRA signalling, this dominant negative mutant would not be able to rescue the phenotype of interest across the four mTKIs. We showed that expression of the gatekeeper mutant receptor in the A204 cell line confers resistance to each of the four mTKIs under study when compared to wildtype (WT) receptor (Fig. 3B), thereby confirming that these drugs act through the same mechanism of action.

Recognising that additional candidate mechanisms of resistance may be present in these cell lines, we undertook gene expression analysis to determine the molecular differences between the parental A204 and G402 cell lines compared with their acquired resistant counterparts. Comparative analysis of transcriptomic profiles identified 10 upregulated and 13 downregulated genes respectively across the mTKI resistant sublines versus the parental cell lines (Figure S4A). In addition, gene set enrichment analysis (GSEA) found that “positive regulation of leukocyte mediated cytotoxicity” and “antigen processing and presentation” were the top pathways that were significantly enriched in the resistant cell lines (Table S3). Interestingly, these genes were involved in antigen presentation and include multiple members of the human leukocyte antigen (HLA) complex, peptide transport and processing proteins (TAP and ERAP2) as well as cytokines (IL12). In contrast, gene sets downregulated in the resistant cell lines were core components of the chromosome separation machinery and RNA splicing (Table S3).

### mTKIs induce temporal collateral sensitivity and resistance to a spectrum of different drugs

**Fig. 4:**
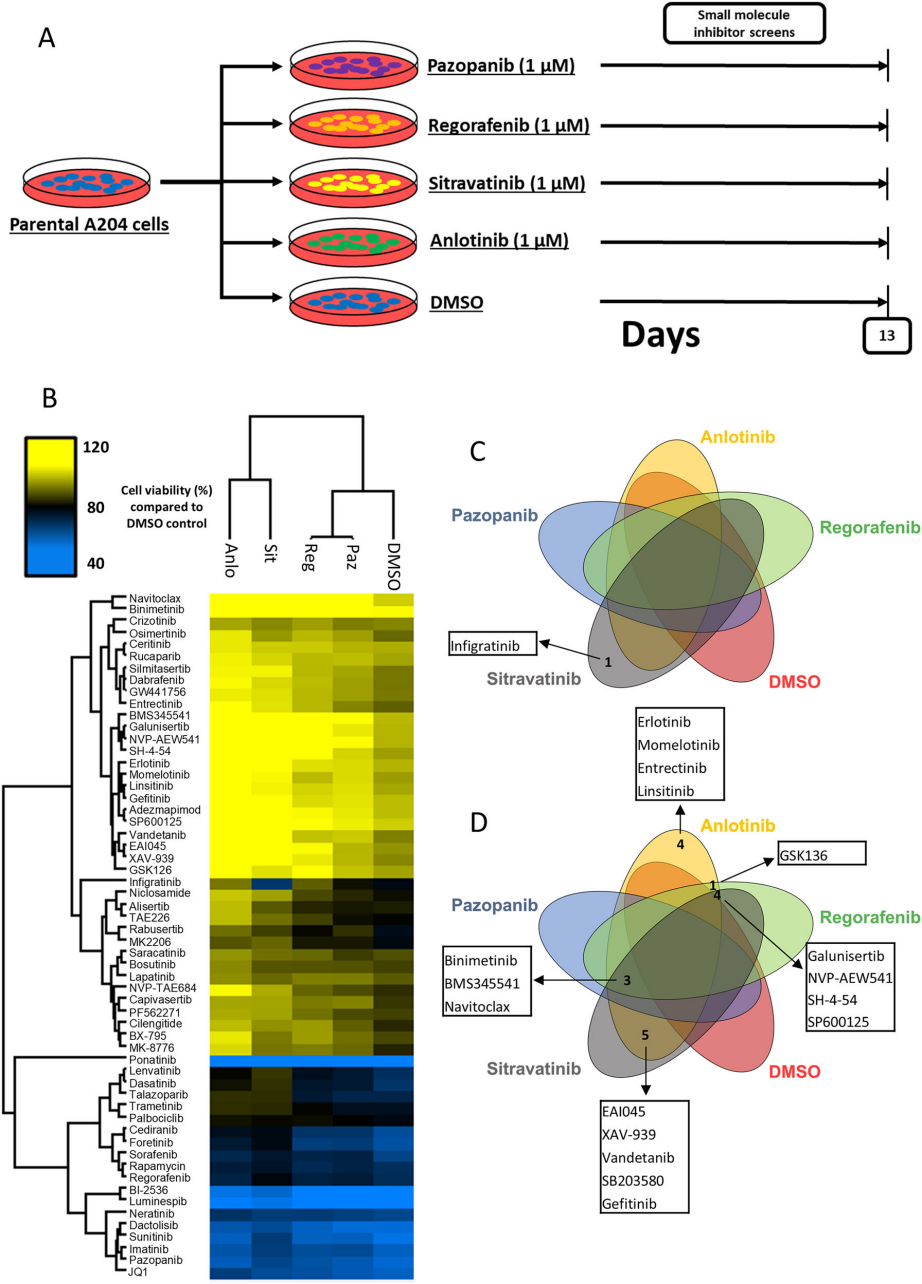
Induction of collateral sensitivity and resistance following pre-treatment with mTKIs. **A** Schematic outlining the experimental protocol for assessing the temporal emergence of collateral sensitivity in pre-treated A204 cells. **B** Small molecule inhibitor screens of A204 parental cells pre-treated with DMSO or indicated mTKIs (0.5 μM) for 13 days. Cell viability was normalised to DMSO control (*n* = 2). Two-way hierarchical clustering based on Euclidean distance was performed by Perseus software. **C** Venn diagram showing the number and identity of inhibitors that display collateral sensitivities ( ≤ 70% cell viability compared to DMSO control) induced by TKI pre-treatment of parental A204 cells. **D** Venn diagram showing the number and identity of inhibitors that display collateral resistance ( ≥ 120% cell viability compared to DMSO control) induced by TKI pre-treatment of parental A204 cells. Paz Pazopanib, Reg Regorafenib, Sit Sitravatinib, Anlo Anlotinib.

Given that these four mTKIs were largely ineffective when used in sequence (Fig. 1G), we sought to determine if other small molecule inhibitors could be used as potential salvage therapies following treatment with mTKIs. A204 parental cells were pre-treated with one of the four mTKIs (or DMSO control) for 13 days prior to being subjected to a panel of 58 small molecule inhibitors designed to target a broad range of oncogenic pathways (Fig. 4A). Details of the panel of inhibitors used are provided in the Supplemental methods. Hits from the screen revealed new collateral sensitivity and resistance phenotypes that were not present in the cells pre-treated with DMSO (Fig. 4B–D). Some of these phenotypes were shared between 2 or more mTKIs, for instance pre-treatment with anlotinib, regorafenib and sitravatanib but not pazopanib induced collateral resistance to multiple inhibitors including galunisertib (TGFβ receptor inhibitor), NVP-AEW541 (IGF-1R inhibitor), SH-4-54 (STAT3/5 inhibitor) and SP600125 (JNK inhibitor) (Fig. 4D). Other hits were specific to particular mTKIs (Fig. 4C, D). One example is pre-treatment with sitravatinib which induced a collateral sensitivity to the FGFR inhibitor infigratinib^34^ at 13 days (Fig. 4B, C). These findings demonstrate that although cells that have achieved acquired resistance to mTKIs share many biological features and display cross resistance (Figs. 2, 3), en route to acquiring drug resistance, new temporal vulnerabilities can emerge which may be exploited for sequential therapy.

**Fig. 5:**
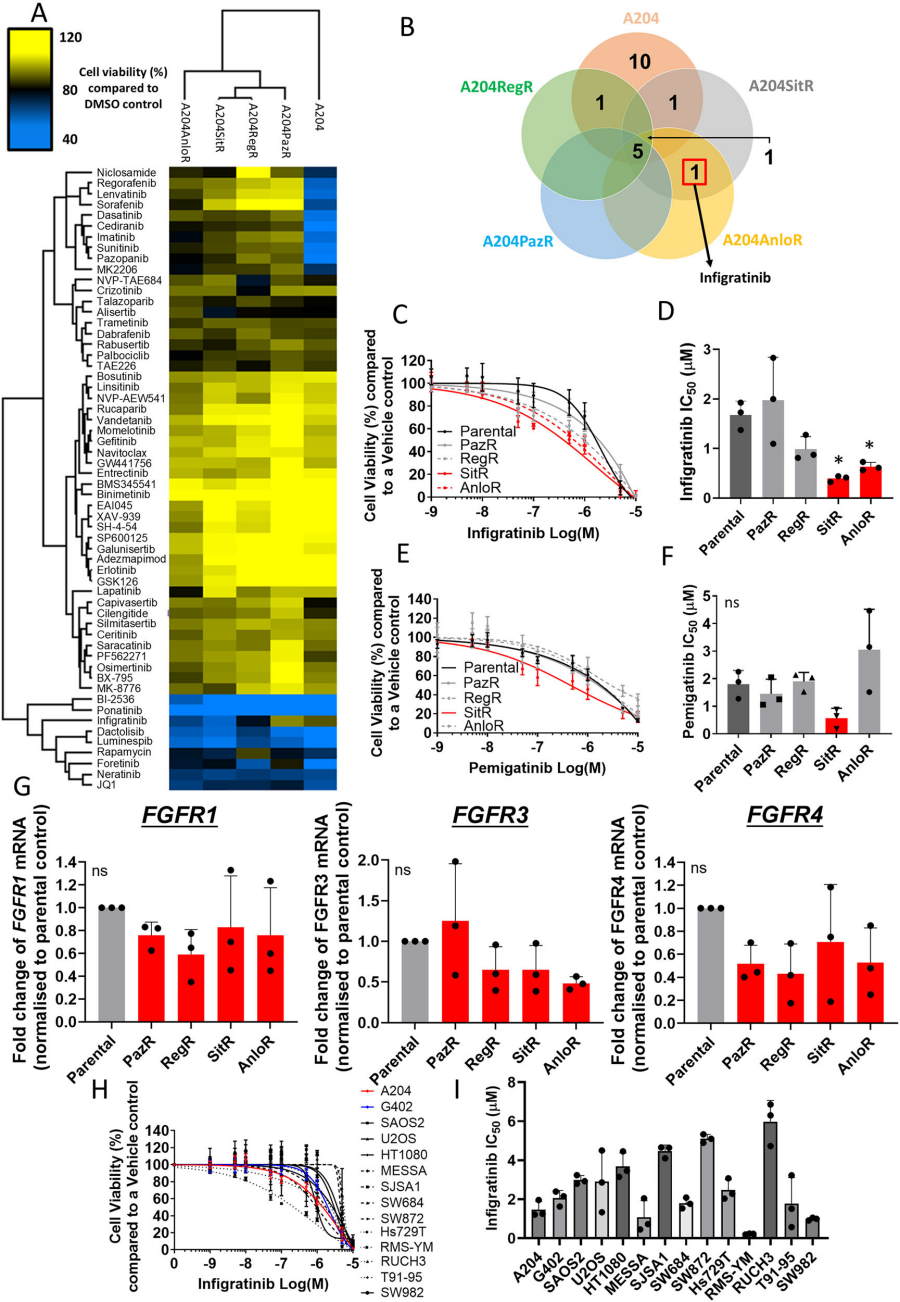
Collateral sensitivity assessment in A204 mTKI-resistant sublines. **A** Small molecule inhibitor screens of A204 and TKI-resistant sublines at an inhibitor concentration of 0.5 μM. Cell viability was normalised to DMSO control (*n* = 2). Two-way hierarchical clustering based on Euclidean distance was performed by Perseus software. **B** Venn diagram showing the number and identity of inhibitors that are effective ( ≤ 70% cell viability compared to DMSO control) at reducing the viability of parental A204 and/or TKI-resistant sublines. **C** Dose response assays of A204 parental and TKI-resistant sublines treated for 72 h with increasing concentrations of infigratinib (Inf) to determine IC_50_ values. Error bars represent standard deviation (*n* = 3). **D** Bar plots displaying IC_50_ values for A204 parental and mTKI-resistant sublines. Cell viability was normalised to DMSO control. Error bars represent standard deviation (*n* = 3). Statistical analysis was undertaken by one-way ANOVAs with Dunnett’s multiple comparison tests (parental A204 control) (* = *p* ≤ 0.05). **E** Dose response assay of A204 parental and TKI-resistant sublines treated for 72 h with increasing concentrations of pemigatinib to determine IC_50_ values. Error bars represent standard deviation (*n* = 3). **F** Bar plots displaying pemigatinib IC_50_ values for A204 parental and mTKI-resistant sublines. Cell viability was normalised to DMSO control. Error bars represent standard deviation (*n* = 3). Statistical analysis was undertaken by one-way ANOVAs with Dunnett’s multiple comparison tests (parental A204 control) (ns = *p* > 0.05). **G** qPCR data displaying the fold change of *FGFR1*, *FGFR3* and *FGFR4* mRNA, normalised to A204 parental cells (*n* = 3). Statistical analysis was undertaken using one-way ANOVAs with Tukey’s multiple comparison tests. There was no significant difference across all cell lines (ns = *p* > 0.05). **H** Dose response assay of a panel of 14 sarcoma cell lines treated for 72 h with increasing concentrations of infigratinib to determine IC_50_ values. **I** Bar plots displaying infigratinib IC_50_ values for the panel of 14 sarcoma cell lines. Error bars represent standard deviation (*n* = 3).

We further assessed if the observed collateral sensitivity to infigratinib was induced in the A204SitR resistant cells where terminal acquired resistance had been achieved. By subjecting the four acquired resistant cell lines to the panel of 58 small molecule inhibitors, we determined drug resistance or sensitivity profiles to infer subsequent lines of treatment following acquired mTKI resistance (Fig. 5A). Analysis of drug hits from the targeted panel showed that only infigratinib fulfilled the criteria of collateral sensitivity where it was ineffective in the parental A204 cells but reduced the cell viability of A204SitR and to a lesser extent A204AnloR (Fig. 5B–D). This trend was also observed in a second structurally distinct pan-FGFR inhibitor pemigatinib^35^ (Fig. 5E, F), although this effect was not statistically significant. This collateral sensitivity was not due differences in gene expression levels of *FGFR1*, *FGFR3* and *FGFR4* (A204 cells do not express *FGFR2*^36^) between A204SitR and the other resistant sublines (Fig. 5G). Infigratinib collateral sensitivity was also observed in G402SitR but not the other G402 mTKI resistant sublines (Figure S5A, B). The G402SitR cell line showed an upregulation of *FGFR1* levels compared to the parental cell line but not *FGFR3* or *FGFR4* (Figure S5C).

We then evaluated whether sarcoma cell lines with primary resistance to sitravatinib harboured intrinsic sensitivity to infigratinib and subjected the 14 sarcoma cell line panel to infigratinib treatment. We show that across the panel, only the rhabdomyosarcoma sarcoma cell line RMS-YM harboured profound sensitivity to infigratinib (Fig. 5H, I). This finding is consistent with previous reports that show that infigratinib sensitivity in this cell line is due to amplification of 8p11 which contains *FGFR1*^37,38^. Our data indicate that the sensitivity to the FGFR inhibitor infigratinib is not a general feature of sarcoma cell lines that exhibit primary resistance to mTKIs but rather a consequence of acquired resistance to sitravatinib.

**Fig. 6:**
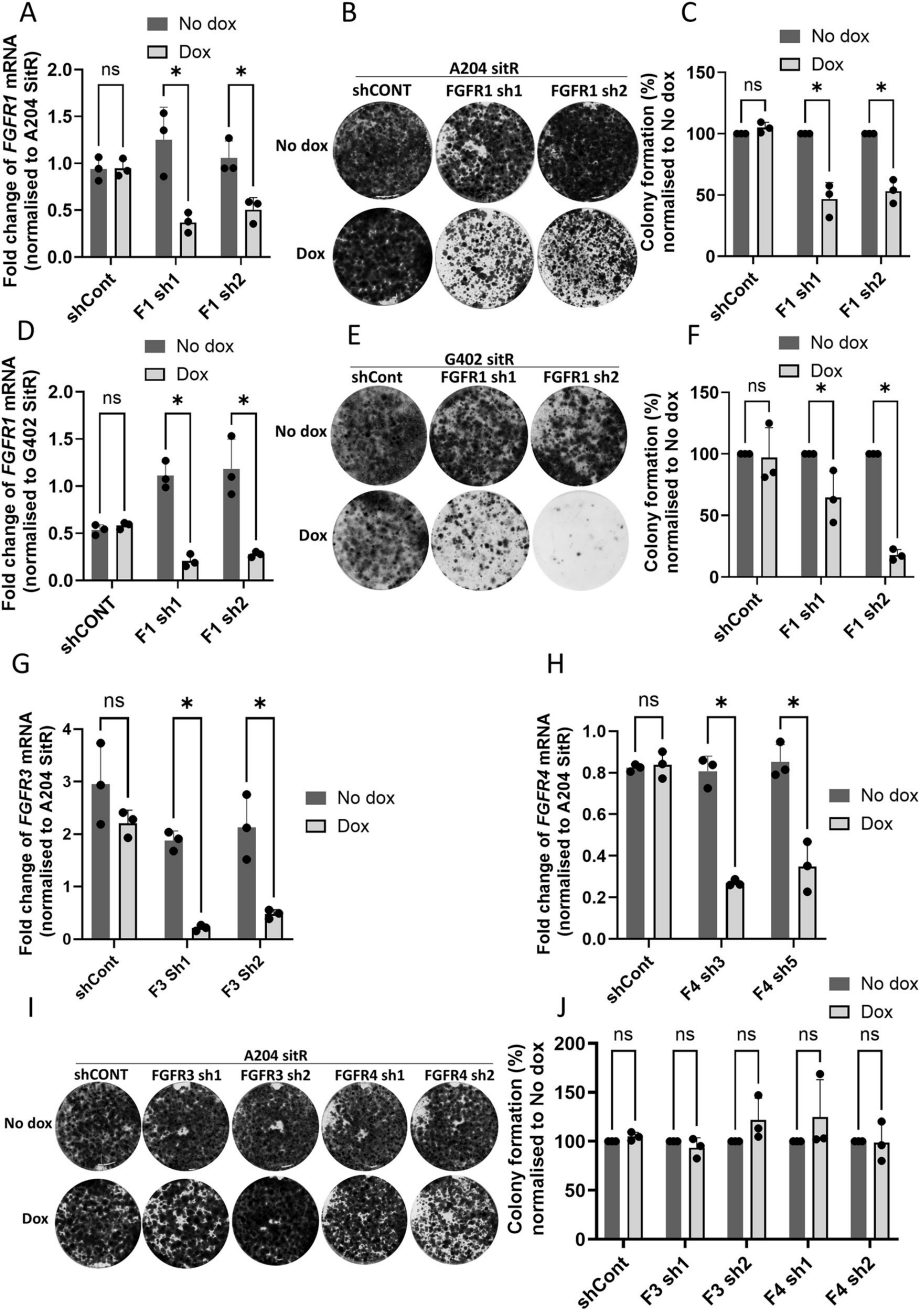
Infigratinib collateral sensitivity is attributed to FGFR1 expression. **A** qPCR data displaying the fold change of *FGFR1* in A204SitR cells transduced with either shControl (shCONT) or shRNA sequences targeting FGFR1 (F1 sh1 and F1 sh2) and treated with (dox) or without (no dox) 2 μg/ml doxycycline. mRNA levels normalised to A204SitR (*n* = 3). **B** Colony formation assays of A204SitR transduced with shCont or two shRNA sequences targeting FGFR1 (F1 sh1 and F1 sh2). Cell lines treated with (dox) or without (no dox) 2 μg/ml doxycycline over a period of 2 weeks. Images are representative of 3 separate experiments. **C** Quantification of A204SitR colony formation assays normalised to shCONT (*n* = 3). **D** G402SitR qPCR data displaying the fold change of *FGFR1* in G402SitR cells transduced with shCONT or two shRNA sequences targeting FGFR1 (F1 sh1 and F1 sh2). mRNA levels normalised to G402SitR (*n* = 3). **E** Colony formation assays of G402SitR transduced with shControl or two shRNA sequences targeting FGFR1 (F1 sh1 and F1 sh2) and treated with (dox) or without (no dox) 2 μg/ml doxycycline over a period of 2 weeks. Images are representative of 3 separate experiments. **F** Quantification of G402SitR colony formation assays normalised to shCONT (*n* = 3). Statistical analysis was undertaken by Student’s unpaired T tests (* = *p* ≤ 0.05). **G** qPCR data displaying the fold change of *FGFR3* in A204SitR cells transduced with either shControl (shCONT) or shRNA sequences targeting FGFR3 (F3 sh1 and F3 sh2) and treated with (dox) or without (no dox) 2 μg/ml doxycycline for a period of 72 h. mRNA levels normalised to A204SitR (*n* = 3). **H** qPCR data displaying the fold change of *FGFR4* in A204SitR cells transduced with either shControl (shCONT) or shRNA sequences targeting FGFR4 (F4 sh1 and F4 sh2) and treated with (dox) or without (no dox) 2 μg/ml doxycycline for a period of 72 h. mRNA levels normalised to A204SitR (*n* = 3). Statistical analysis was undertaken by Student’s unpaired T tests (* = *p* ≤ 0.05). **I** Colony formation assays of A204SitR transduced with shCont or two shRNA sequences targeting FGFR3 and FGFR4. Cell lines treated with (dox) or without (no dox) 2 μg/ml doxycycline for a period of 2 weeks. Images are representative of 3 separate experiments. **J** Quantification of A204SitR colony formation assays normalised to shCONT (*n* = 3). Statistical analysis was undertaken by Student’s unpaired T tests (no significant differences ns).

To confirm the involvement of FGFR signalling in the sitravatinib resistant cell lines, we undertook genetic depletion of *FGFR1* using doxycycline inducible short hairpin RNAs (shRNAs). Use of two different shRNA constructs resulted in a reduction in the colony formation potential in both the A204SitR and G402SitR cell lines (Fig. 6A–F). Notably, when similar experiments were repeated with shRNAs against *FGFR3* and *FGFR4* (A204 and G402 cell lines do not express *FGFR2*), there was no reduction in colony formation (Fig. 6G–J), confirming that the collateral sensitivity is specific to FGFR1 and not the other members of the FGFR family.

### Sitravatinib treatment confers collateral sensitivity to infigratinib

**Fig. 7:**
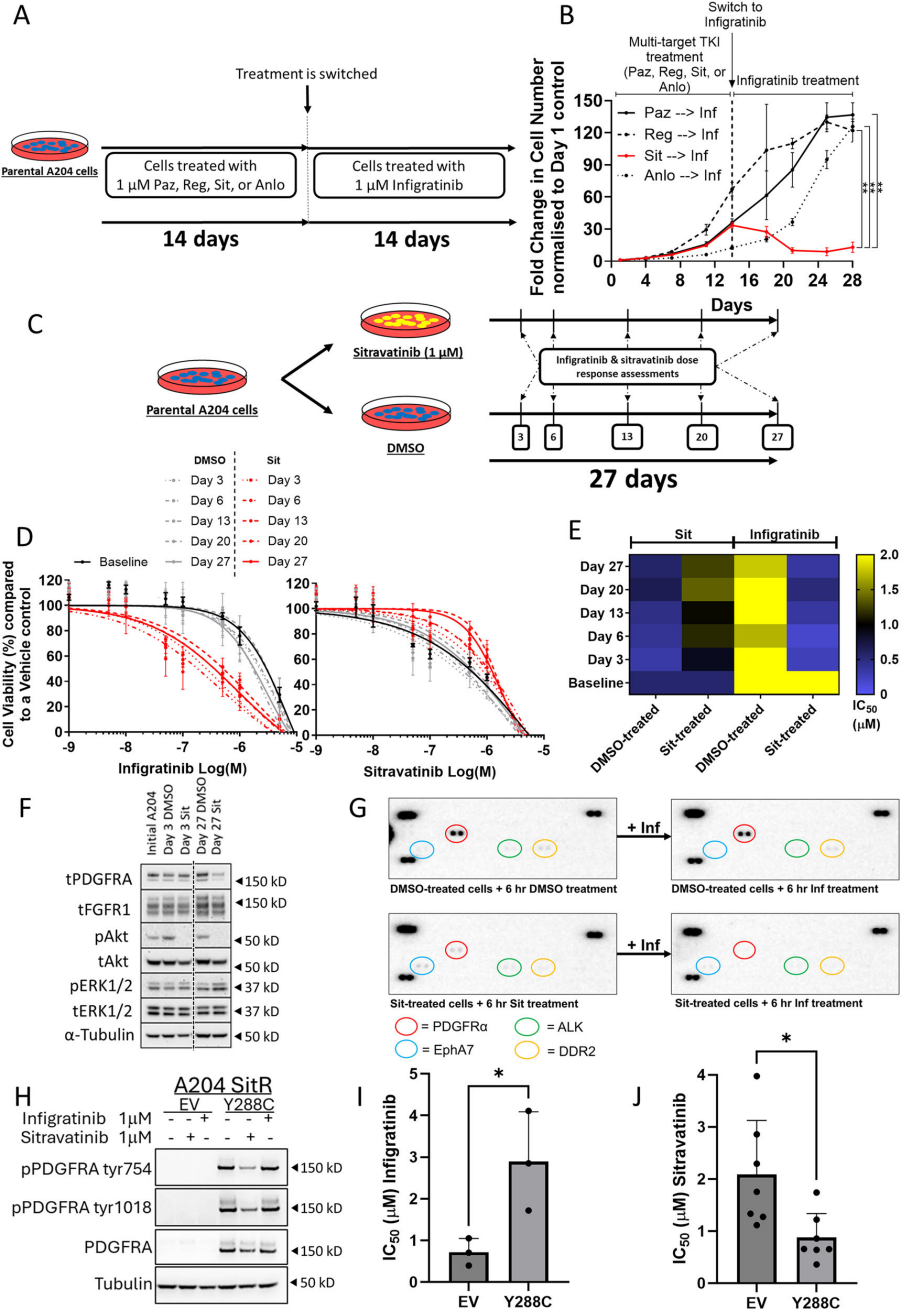
Temporal assessment of infigratinib collateral sensitivity in A204 sitravatinib pre-treated cells. **A** Schematic outlining the experimental protocol for assessing the emergence of infigratinib collateral sensitivity in mTKI pre-treated A204 cells. **B** Growth curve assays of A204 cells to measure the fold change in cell number over a period of 4 weeks in cells treated initially with 1 μM mTKIs for 2 weeks before switching to infigratinib (1 μM) for a further 2 weeks. Fold change was normalised to day 1 control (*n* = 2). Error bars represent standard deviation. Statistical analysis was undertaken using one-way ANOVAs with Tukey’s multiple comparison tests (**= *p* ≤ 0.01). **C** Schematic outlining the experimental protocol for assessing the temporal emergence of infigratinib collateral sensitivity in sitravatinib pre-treated A204 cells. **D** Dose response assays of A204 parental cells following treatment as outlined in schematic C to determine IC_50_ values. Cell viability was normalised to DMSO control. Error bars represent standard deviation (*n* = 2). **E** Heatmap of the IC_50_ values for dose response assays outlined in schematic C (*n* = 2). **F** Immunoblot mapping alterations in PDGFRA, FGFR1, Akt, and ERK1/2 expression and phosphorylation levels in cells treated with either DMSO or 1 μM sitravatinib at 3 or 27 days. Image is representative of two separate experiments. **G** Phospho-RTK array was performed upon A204 cells that had either been pre-treated with 14 days of DMSO or 1 μM sitravatinib. After 14 days, cells were then either continued on their respective treatment (DMSO or 1 μM sitravatinib) for 6 h or switched to 1 μM infigratinib for 6 h. Spots in the top left/right and bottom left of the array are reference spots. RTKs of interest are circled and colour-coded. Image is representative of a single experiment (*n* = 1). Paz Pazopanib, Reg Regorafenib, Sit Sitravatinib, Anlo Anlotinib, Inf Infigratinib. **H** Immunoblot of PDGFRA expression and phosphorylation levels in A204SitR expressing empty vector (EV) or constitutively active (Y288C) PDGFRA, treated for 6 h with either DMSO, infigratinib or sitravatinib. Image is representative of two separate experiments. **I** Bar plots displaying infigratinib IC_50_ values after 72 h treatment for A204SitR transduced with EV or Y288C PDGFRA. Error bars represent standard deviation (*n* = 3). **J** Bar plots displaying sitravatinib IC_50_ values after 72 h treatment for A204SitR transduced with EV or Y288C PDGFRA. Error bars represent standard deviation (*n* = 7). Statistical analysis was undertaken by Student’s unpaired T tests (* = *p* ≤ 0.05).

We validated the sitravatinib hit from the screen by pre-treating the parental A204 cells with each of the four mTKIs for 14 days, following which, the drug was switched to infigratinib, and cell growth followed for another 14 days (Fig. 7A). Only pre-treatment with sitravatinib led to a reduction in cell growth upon switching to infigratinib treatment, highlighting the drug specific nature of the observed temporal collateral sensitivity (Fig. 7B). A similar phenotype was observed in the G402 cell line (Figure S6). In order to ascertain how early following sitravatinib treatment this collateral sensitivity was induced, A204 cells were pre-treated with either sitravatinib or DMSO control for 3, 6, 13, 20 and 27 days and then subjected to infigratinib or sitravatinib (as a control) dose response assessment (Fig. 7C). We found that collateral sensitivity was rapidly induced as early as 3 days following exposure to sitravatinib (Fig. 7D, E). Western blot analysis across these timepoints found that continuous treatment with sitravatinib led to a time-dependent reduction in PDGFRA expression levels and a reduction in pAKT but no alterations in the protein expression levels of FGFR1 when compared to DMSO control treatment (Fig. 7F).

To evaluate the global effects on RTK phosphorylation levels upon treatment with sitravatinib and infigratinib in A204 cells, we undertook RTK antibody array analysis. Cells were treated with either DMSO or sitravatinib for 14 days prior to switching to either the same treatment or infigratinib for 6 h (Fig. 7G). Sitravatinib led to a decrease in PDGFRA phosphorylation compared to DMSO. Notably there was still residual PDGFRA phosphorylation in the A204 cells in the presence of sitravatinib. However, upon subsequent treatment with infigratinib, there was a complete abrogation of PDGFRA phosphorylation. In keeping with our published observations that genetic silencing of PDGFRA reduces parental A204 cell viability^32^, our data suggest that complete suppression of PDGFRA activity by sequential treatment of sitravatinib following by infigratinib is likely to be responsible for the observed temporal collateral sensitivity to this drug.

We sought to investigate if PDGFRA activity contributes to the observed sensitivity to infigratinib by transducing the A204SitR cell line with a constitutively active mutant of PDGFRA (Y288C)^39^. We show that expression of the PDGFRA Y288C mutant not only sensitised the cells to sitravatinib treatment, it also led to a corresponding induction of infigratinib resistance (Fig. 7H–J). These data confirms that 1. the loss of PDGFRA expression is the primary mechanism of induced sitravatinib resistance in these cells, and 2. suppression of PDGFRA activity contributes to the mechanism by which collateral sensitivity to infigratinib is achieved.

## Discussion

Patients with non-adipocytic STS are currently treated with the mTKI pazopanib following failure of prior anthracycline therapy^3,25^. However, the median duration of response is short, and a significant subset of patients do not benefit from this drug^25^. Other mTKIs that have been evaluated in this setting include regorafenib and anlotinib which show similar results in clinical trials and real-world data^27,40,41^. There is therefore an urgent need to identify ways to tackle resistance to this class of drugs and achieve deeper and more durable responses in these patients. Using malignant rhabdoid tumour cell lines as a model system, here we show that cancer cells that acquire resistance to mTKIs are cross resistant to each other and share several molecular and phenotypic features. Consequently, sequential treatment with these mTKIs does not achieve further benefits in suppressing cancer cell growth. Even with the most potent of the four mTKIs (anlotinib), this approach only leads to improvements that are at best incremental. These data suggest that using any of these drugs for salvage therapy following mTKI failure would not be optimal. However, en route to the acquisition of resistance, these cells acquire new drug vulnerabilities that can be harnessed to achieve durable responses in vitro. We propose that exploiting collateral sensitivity for salvage and adaptive therapy is a therapeutic approach that should be explored in future STS clinical trial designs.

Collateral sensitivity is a concept in which new vulnerabilities arise as a result of the fitness costs associated with the acquisition of drug resistance^17,21^. In the context of TKIs, this approach has thus far only been studied in cancer types that are addicted to mutant oncogenic kinases such as BCR-ABL, EML4-ALK and mutant EGFR^19,22,23^. For instance, Zhao et al. showed that acquisition of on-target mutations in BCR-ABL as a result of exposure to BCR-ABL-targeted inhibitors led to temporal collateral sensitivity to non-classical BCR-ABL inhibitors such as crizotinib and foretinib at intermediate stages of evolution^23^. Similarly, Acar et al. determined that lung cancer cells that harbour oncogenic mutant EGFR that acquire resistance to the EGFR inhibitor gefitinib develop collateral sensitivity to c-MET inhibitor capmetinib^19^. In contrast, in cancer types such as renal cell carcinoma, hepatocellular carcinoma, colorectal cancer and STS where mTKIs are routinely used as standard of care, there is no clear oncogenic driver kinase and the mechanisms of mTKI activity in patients remain to be fully defined. Our study demonstrates, for the first time, that collateral sensitivity can be exploited beyond the paradigm of mutant oncogenic driver kinases with a broader utility in cancers such as STS.

Our study finds that pre-treatment of cells with mTKIs can induce collateral sensitivity and resistance to a range of different drugs, some of which are shared and others unique. In particular, we show that the mTKI sitravatinib induces collateral sensitivity to the FGFR inhibitor infigratinib in two different rhabdoid tumour cell lines. Given the broad-spectrum nature of these drugs, the reasons for the differential induction of collateral sensitivity and resistance between the four mTKIs are unknown. Although a direct head-to-head comparison of the target selectivity profile between sitravatinib and the other three mTKIs used in this study has not been undertaken, among the key differences in sitravatinib is its ability to target the TAM receptors (Tyro, Axl and Mer)^28,29^ which may be responsible for the distinct collateral sensitivity profile. We further find that while sitravatinib substantially reduces PDGFRA phosphorylation in A204 cells, it does not abolish its activity. This observation is consistent with recently reported reverse phase protein array data from a liposarcoma patient treated with sitravatinib^30^. We and others have previously shown that the PDGFRA and FGFR1 RTKs are important for the survival of malignant rhabdoid tumour cells and complete suppression of PDGFRA is necessary to achieve cell death^32,42^. Following on from sitravatinib treatment, we show that sequential treatment with infigratinib completely abrogates PDGFRA phosphorylation. Infigratinib is a selective FGFR inhibitor with no reported cross inhibitory activity with the PDGFR family of receptors^34^. RTK co-activation is a known mechanism of TKI drug resistance^43,44^ and it is possible that crosstalk between these PDGFR and FGFR RTKs may explain why FGFR inhibition can result in the concomitant decrease in PDGFRA phosphorylation. Future work will determine if this RTK crosstalk is responsible for the induction of FGFR inhibitor sensitivity by sitravatinib treatment in rhabdoid tumour cells.

Our findings indicate that infigratinib is a candidate for drug repurposing in either the salvage or adaptive therapy setting following treatment with sitravatinib. In a phase II trial of sitravatinib in well differentiated/dedifferentiated liposarcoma patients (*n* = 29), although 41% of patients were progression free at 12 weeks, the confirmed overall response rate was 0%^30^, indicating that a substantial proportion of patients had intrinsic or acquired resistance to this drug. With the known toxicities associated with drug combinations^45^, sequential treatments are an attractive alternative to achieve durable clinical benefit. This approach has been clinically proven with selected molecular subtypes of lung cancers and gastrointestinal stromal tumours where sequential treatments with up to fourth line TKIs have led to clinically meaningful improvements in patient survival^8–12^. Our data provide evidence for the rationale use of FGFR inhibitors upon failure of sitravatinib. Furthermore, since infigratinib sensitivity was brought about rapidly within 3 days of exposure, even before terminal drug resistance is achieved, FGFR inhibitors can also be considered as a maintenance therapy following initial treatment with sitravatinib in patients with STS.

There are several limitations to our study. We have only investigated a small number of mTKIs in this study and our findings may not be generalisable for the broader class of drugs. In addition, STS comprise more than 80 different histological subtypes with distinct biology and our data on malignant rhabdoid tumours need to be validated in other histotypes. Several of the mTKIs are known to have anti-angiogenic properties and our experiments have focused on tumour cell intrinsic drug profiles which do not consider the influence of tumour microenvironmental factors such as the vasculature. Future experiments incorporating microenvironmental components are required. Sarcomas are known to be heterogeneous tumours and the use of cell lines is unlikely to represent the complete spectrum of clones present in a heterogeneous population within a tumour. Each of these clones may have unique evolutionary trajectories and observed collateral sensitivities/resistance. Similar efforts incorporating the use of cellular barcoding approaches with more heterogeneous patient-derived models would be needed to address this limitation^46^. Our experiments also suggest that a personalised approach towards screening is necessary to identify specific collateral sensitivities in individual cell lines, whether this can be translated at scale to sarcoma patients in general requires further research. Finally, we only evaluated a small panel of small molecule inhibitors to identify new vulnerabilities, with the choice primarily driven by prior knowledge of signal transduction pathways and mechanisms of action. Incorporation of a more comprehensive panel of compounds such as the Prestwick Chemical Library of 1280 FDA-approved compounds may facilitate identification of new vulnerabilities with clinical utility. Despite these limitations, our studies provide new avenues to tackle drug resistance to mTKIs, a current clinical unmet need, and demonstrates how the use of drug repurposing can lead to new opportunities in personalised adaptive treatment strategies to attain more durable benefits for patients with STS.

## Methods

### Cell lines

A204 and G402 cell lines were obtained from ATCC. All other cell lines except for HEK293T were a gift from Professor Janet Shipley. HEK293T was a gift from Professor Chris Lord. Cell lines were cultured in DMEM (A204, G402, SAOS2, U2OS, HT1080, MESSA, SW684, SW872, Hs729T, RUCH3, T91-95, SW982 and HEK293T) or RPMI 1640 media (SJSA1 and RMS-YM), supplemented with 10% foetal bovine serum (FBS) (Gibco) and 0.5% penicillin/streptomycin. Cell lines were frozen in 10% dimethyl sulfoxide (DMSO) (Sigma Aldrich) in FBS. All cells were cultured in 95% air:5% CO_2_ at 37 °C. Media was replenished twice weekly. Details of cell lines used in this study are provided in the Supplemental methods. Cell lines were tested for mycoplasma contamination using MycoStrip detection kit (InvivoGen).

### Derivation of acquired resistance sublines

The A204 and G402 cell lines were subjected to long-term treatment with pazopanib, regorafenib (both from LC Laboratories), sitravatinib or anlotinib (both from Selleck Chemicals) to derive acquired resistance sublines. Parental A204 and G402 cells were initially grown in media containing one of these four mTKIs at the concentration of the determined inhibitory constant (IC_50_) values from cell viability assays. The drug was increased when the cells had proliferated to near confluency alongside minimal visible cell death. For A204 drug concentration was increased in a stepwise manner to 1 µM, 2 µM and 3 µM, until a final concentration of 5 µM was maintained in A204PazR, A204RegR and A204SitR cells and 3 µM for A204AnloR sublines. G402 drug concentration was increased in a stepwise manner to 0.25 µM, 0.5 µM, 1 µM, 2 µM, 3 µM and 4 µM and a final concentration of 5 µM was maintained for G402PazR and G402RegR, 4 µM for G402SitR and 1 µM for AnloR. Resistance was confirmed using cell viability and colony formation assays. Media and inhibitors were replenished twice weekly.

### Cell viability assay

Cells (2000/well) were seeded into 96 well plates. After 24 h, cells were treated with inhibitors at the indicated concentrations and incubated for 72 h prior to cell viability measurement by CellTiter-Glo assay (Promega), following the manufacturer’s recommendations. Measurements were undertaken on Victor X5 (PerkinElmer), Spark (Tecan) or FLUOstar Omega (BMG Labtech) plate readers. IC_50_ data were generated from dose response curves using four-parameter regression fit in PRISM (Graphpad). Inhibitors included pemigatinib (MedChemExpress), pazopanib, regorafenib, sitravatinib, anlotinib and infigratinib (Selleck Chemicals).

### Colony formation assay

Cells (10,000/well) were seeded into 6 well plates. After 24 h, cells were treated with inhibitors at the indicated concentrations for a duration of 2 weeks. Media and inhibitors were replenished every 72 h. After 2 weeks, cells were fixed using Carnoy’s fixative (3:1 methanol:acetic acid) and stained with 1% crystal violet solution (Sigma Aldrich). Plates were digitally imaged using ChemiDoc Touch Imaging System (Bio-Rad) or G-Box Chemi-XX6 (Syngene) imagers. Densitometry analysis was performed using the ImageJ software program (National Institutes of Health).

### Apoptosis assay

Cells (2000/well) were seeded into 96 well plates. After 24 h, cells were treated with inhibitors at the indicated concentrations and incubated for 24 h prior to apoptosis measurement by Caspase-Glo 3/7 assay (Promega), following the manufacturer’s recommendations. Measurements were undertaken on Victor X5 (PerkinElmer) or FLUOstar Omega (BMG Labtech) plate readers.

### Immunoblotting

After the indicated time period post-treatment, cells were lysed in radioimmunoprecipitation assay (RIPA) buffer supplemented with protease and phosphatase inhibitors (Thermo Scientific) at 4 °C. Lysates were loaded onto NuPAGE Novex 4–12% Bis-Tris gels (Invitrogen), followed by blotting onto iBlot PVDF membranes (Invitrogen). The following primary antibodies were used: rabbit anti-pAkt (S473) (1:1000; 193H12; Cell Signalling Technology; 4058), rabbit anti-Akt (pan) (1:1000; C67E7, Cell Signalling Technology; 4691), rabbit anti-phospho p44/42 MAPK (phosphoERK1/2) (T202/Y204) (1:1000; D13.14.4E; Cell Signalling Technology; 4370), rabbit anti-p44/42 MAPK (ERK1/2) (1:1000; 137F5; Cell Signalling Technology; 4695), rabbit anti-pPDGFRA (Y754) (1:500; 23B2; Cell Signalling Technology; 2992), rabbit anti-pPDGFRA (Y1018) (1:500; Cell Signalling Technology; 4547), rabbit anti-PDGFRA (1:1000; D1E1E; Cell Signalling Technology; 3174), rabbit anti-FGFR1 (1:1000; EPR806Y; Abcam; ab76464) and mouse anti-α-tubulin (1:5000; Sigma Aldrich; T5168). Secondary antibodies were horseradish peroxidase (HRP)-conjugated secondary antibodies (HRP-conjugated mouse [SignalChem; G32-62G-1000] or rabbit [Cell Signalling Technology; 7074]). Additional details of immunoblotting protocol are provided in Supplemental methods.

### Small molecule inhibitor screen

Cells (2000/well) were seeded into 96 well plates. After 24 h, cells were treated with inhibitors at 0.5 µM and incubated for 72 h prior to cell viability measurement by CellTiter-Glo assay (Promega), following the manufacturer’s recommendations. Measurements were undertaken on Victor X5 plate readers (PerkinElmer). A detailed list of the small molecule inhibitors utilised along with their primary target(s) and supplier is presented in the Supplemental methods. Data were clustered two-way based on Euclidean distance using the Perseus software^47^.

For temporal analysis, A204 cells (1,000,000/flask) were seeded into T75 flasks. After 24 h, the T75s were stratified to receive either DMSO (Sigma Aldrich) or 1 µM of either pazopanib, regorafenib, sitravatinib or anlotinib. Media and inhibitors were replenished twice weekly. Cells were maintained at between 70 and 90% confluency. After 13 days of treatment, cells were seeded (2000/well) into 96 well plates and subjected to the small molecule inhibitor panel.

### Growth curve and sequential treatment assay

Cells (500/well) were seeded into black-walled 96 well plates. After 24 h, one plate was fixed with 10% neutral-buffered formalin solution (Sigma Aldrich) and stored at 4 °C. The remaining plate sets were treated with DMSO (Sigma Aldrich) or 1 µM of indicated inhibitor for a period of six weeks. For the sequential treatment assay, after an initial treatment period of two weeks (or two and a half weeks for G402), the administered inhibitor was altered as indicated. Media and inhibitors were replenished every 72 h. Fixed cells were stained with Hoechst 33342 (R & D Systems) for 10 mins at 37 °C, followed by phosphate-buffered saline (PBS) washes. Direct cell count was undertaken using a Celigo Image Cytometer (Nexcelcom BioScience). Cell count for a particular inhibitor sequence was ceased upon reaching confluency.

### Assessment of cell viability and signalling following temporal treatment with sitravatinib and infigratinib

The baseline sensitivities to sitravatinib and infigratinib were evaluated in parental A204 cells (2000/well) via cell viability assays. Using the same T75 flask of parental A204 cells that were used for seeding the baseline sensitivity evaluation, cells were also seeded into two separate T75 flasks (1000,000/flask). After 24 h of seeding, these cells were treated with either DMSO or 1 µM sitravatinib. Cells were allowed to grow for four weeks post-seeding in their respective treatments. Media and inhibitors were replenished twice weekly. Cells were maintained at between 70–90% confluency. After 3, 6, 13, 20 and 27 days of treatment, cells were evaluated for temporal changes in their sensitivities to sitravatinib and infigratinib using cell viability assays and changes in intracellular signalling molecules assessed using immunoblotting.

### Statistics and reproducibility

Statistical analysis were performed using GraphPad Prism 10 software. Unless stated, experiments included at least three independent biological replicates. Data were presented as the mean ± standard deviation (SD), unless stated otherwise. The two-tailed unpaired Student’s *t*-test was used for comparing two groups. One-way ANOVA test was used for comparing more than two groups with Dunnett’s multiple comparison test used when comparing multiple treated samples with a control sample or Tukey’s multiple comparison test when comparing all possible pairwise comparisons in a group. *P  <  0.05, **P  <  0.01, ***P  <  0.001, and ****P  <  0.0001.

Details for phospho-receptor tyrosine kinase (RTK) array, quantitative polymerase chain reaction (qPCR), shRNA FGFR knockdown, PDGFRA WT and mutant expression and RNA sequencing are provided in the Supplemental methods.

### Reporting summary

Further information on research design is available in the Nature Portfolio Reporting Summary linked to this article.

## Supplementary information

Supplementary Information

Supplementary Data

Description of Additional Supplementary Files

Reporting Summary

## Data Availability

Raw RNAseq data have been deposited to GEO (series record GSE298261). All uncropped and unedited blot images are included in Figs. S7–S15. Source data for all graphs can be obtained in supplementary data file. All other data are available from the corresponding author on reasonable request.
