# Supplementary material for: Exploiting collateral sensitivity in the evolution of resistance to tyrosine kinase inhibitors in soft tissue sarcomas
Source: Commun Biol. 2025 Aug 8;8:1185. doi: 10.1038/s42003-025-08652-1 (PMC12334625; doi:10.1038/s42003-025-08652-1)
Supplement: Supplementary file 1 — Supplementary Information [file 42003_2025_8652_MOESM1_ESM.pdf]

Supplemental Tables, Figures & Methods

Table S1. Panel of 14 sarcoma cell lines and their response to multi-target tyrosine kinase inhibitors. Anlo, Anlotinib; Paz, Pazopanib; Reg, Regorafenib; S.D., Standard deviation; Sit, Sitravatinib; IC_{50}, Inhibitory constant; n/a, Not available.

Cell line	Paz IC_{50} (µM) (± S.D.)	Reg IC_{50} (µM) (± S.D.)	Sit IC_{50} (µM) (± S.D.)	Anlo IC_{50} (µM) (± S.D.)
**A204**	0.37 (± 0.10)	0.83 (± 0.16)	0.30 (± 0.06)	0.62 (± 0.18)
**G402**	0.38 (± 0.05)	0.71 (± 0.06)	0.23 (± 0.10)	0.41 (± 0.41)
**SAOS2**	> 10 (n/a)	5.92 (± 0.09)	3.46 (± 0.69)	3.48 (± 0.65)
**U2OS**	> 10 (n/a)	8.97 (± 2.51)	2.38 (± 0.79)	3.27 (± 0.37)
**HT1080**	> 10 (n/a)	> 10 (n/a)	4.01 (± 1.37)	2.67 (± 0.93)
**MESSA**	> 10 (n/a)	> 10 (n/a)	1.73 (± 0.40)	3.84 (± 0.87)
**SJSA1**	> 10 (n/a)	> 10 (n/a)	3.66 (± 0.09)	4.02 (± 0.06)
**SW684**	8.26 (± 0.45)	6.89 (± 2.46)	1.95 (± 0.74)	1.68 (± 0.22)
**SW872**	> 10 (n/a)	> 10 (n/a)	3.57 (± 0.88)	4.50 (± 0.55)
**Hs729T**	> 10 (n/a)	> 10 (n/a)	2.61 (± 1.84)	4.20 (± 0.83)
**RMS-YM**	5.34 (± 1.50)	4.27 (± 0.93)	0.84 (± 0.17)	1.64 (± 0.50)
**RUCH3**	> 10 (n/a)	> 10 (n/a)	2.91 (± 1.23)	4.27 (± 0.74)
**T91-95**	> 10 (n/a)	7.99 (± 0.33)	2.10 (± 0.16)	1.44 (± 0.21)
**SW982**	4.68 (± 0.72)	> 10 (n/a)	1.13 (± 0.15)	3.18 (± 1.40)

Table S2. A204 cells combination indices for A204 drug resistant sublines with the TKIs pazopanib (Paz), regorafenib (Reg), stravatinib (Sit) and anlotinib (Anlo). The combination indices were calculated using IC50 based on the Chou-Talalay method (Chou, Cancer Res. 2010). A combination to be considered synergistic, the combination index must be < 1. CI, combination index, n/a, Not available.

TKI treatment	PazR CI	RegR CI	SitR CI	AnloR CI
Paz+Reg	1.95	2.18	n/a	n/a
Paz+Sit	1.91	n/a	2.29	n/a
Paz+Anlo	1.41	n/a	n/a	2.31
Reg+Sit	n/a	1.60	2.43	n/a
Reg+Anlo	n/a	2.60	n/a	2.37
Sit+Anlo	n/a	n/a	2.06	2.18

Table S3. Gene set enrichment analysis of A204 and G402 drug resistant models compared to parental cell lines. Gene set enrichment analysis was performed using Gene Ontology Biological Pathways gene sets. Multiple testing correction was performed using the Benjamini-Hochberg method, at a false discovery rate (FDR) threshold of 0.01. Included genes refers to the number of genes from the gene set contributing to the normalised enrichment score, while set size refers to the total number of genes in the dataset. NES: normalised enrichment score.

Description	Normalised enrichment score	FDR q-value	Included genes	Set size	Genes in dataset
Positive regulation of leukocyte mediated cytotoxicity	1.87	7.15e-4	38	68	HLA-A, FADD, HLA-C, HLA-F, HLA-B, LAMP1, TAP2, B2M, IL12A, LAG3, HLA-DRA, HLA-DRB1, ULBP2, IL18RAP, ARG1, NOS2, IL12RB1, MR1, HLA-H, F2RL1, ULBP3, RAET1E, STX7, RAET1L, CRTAM, IL23A, CD160, RASGRP1, RAET1G, VAV1, CD226, NCR3, IL12B, MICB, KLRC1, HLA-E, KLRK1, PVR
Syncytium formation	1.90	7.38e-4	34	66	EHD1, ADAM9, CAPN2, DYRK1B, ERCC1, GDF15, TCTA, SBNO2, CFLAR, NPHS1, IL4R, CD9, RAPGEF3, NOS1, CXCL10, ADAMTS15, ITGB1, ADGRB1, KCNH1, CD81, RIPOR2, CD53, CAV3, ERVW-1, SH3PXD2A, ERVFRD-1, DOCK5, PTGFRN, EHD2, MYMX, PLEKHO1, ADGRB3, VASH2, MYH9
Syncytium formation by plasma membrane fusion	1.87	7.83e-4	33	62	EHD1, ADAM9, CAPN2, DYRK1B, GDF15, TCTA, SBNO2, CFLAR, NPHS1, IL4R, CD9, RAPGEF3, NOS1, CXCL10, ADAMTS15, ITGB1, ADGRB1, KCNH1, CD81, RIPOR2, CD53, CAV3, ERVW-1, SH3PXD2A, ERVFRD-1, DOCK5, PTGFRN, EHD2, MYMX, PLEKHO1, ADGRB3, VASH2, MYH9
Cell-cell fusion	1.87	7.83e-4	33	62	EHD1, ADAM9, CAPN2, DYRK1B, GDF15, TCTA, SBNO2, CFLAR, NPHS1, IL4R, CD9, RAPGEF3, NOS1, CXCL10, ADAMTS15, ITGB1, ADGRB1, KCNH1, CD81, RIPOR2, CD53, CAV3, ERVW-1, SH3PXD2A, ERVFRD-1, DOCK5, PTGFRN, EHD2, MYMX, PLEKHO1, ADGRB3, VASH2, MYH9
Antigen processing and presentation of endogenous peptide antigen	1.85	0.003	16	25	HLA-A, TAP1, HLA-C, HLA-F, TAPBP, HLA-B, TAP2, ERAP2, B2M, HLA-DRA, HLA-DRB1, ULBP2, HLA-H, ULBP3, RAET1E, RAET1L
Positive regulation of T cell mediated cytotoxicity	1.85	0.004	18	39	HLA-A, FADD, HLA-C, HLA-F, HLA-B, TAP2, B2M, IL12A, HLA-DRA, HLA-DRB1, ULBP2, IL12RB1, MR1, HLA-H, ULBP3, RAET1E, STX7, RAET1L
Regulation of collagen metabolic process	1.84	0.006	24	43	LARP6, CST3, ENG, UCN, SCX, CIITA, SERPINB7, IHH, NOTCH1, ITGA2, ITGB1, MIR149, FAP, INHBA, CREB3L1, WNT4, SERPINF2, F2R, MFAP4, SUCO, MIR29A, RGCC, RUNX1, IL6
Mitochondrial gene expression	-2.28	7.54e-9	119	160	MTERF4, NSUN3, MRPL27, MRPL49, TRMT61B, MRPL54, IARS2, MRPS5, MRPL43, MALSU1, FOXO3, MRPS28, MTRF1L, TRNT1, MRPS11, MRPL12, MTIF3, MRPS16, MRPL21, PTCD1, MRPL55, GADD45GIP1, MRPL34, MRPS35, MTIF2, TEFM, MRPL9, MRPL50, MRPS18C, MRPL42, PPARGC1B, RMND1, UQCC2, GATC, TACO1, MRPL40, MTERF1, TRMT5, TWNK, MRPL32, PNPT1, MRPL16, MRPL44, MTG2, MRPS14, MRPL15, FASTKD2, RCC1L, MTPAP, MRPL45, MRPS17, FASTKD1, MRPS26, MRPS7, TARS2, MRPL35, WARS2, THAP11, TRMT10C, ELAC2, MRPS27, MRPS22, HSD17B10, LRPPRC, FASTKD3, MRPL47, MRPS9, MRPL23, METTL4, MRPS2, CHCHD10, MRPL4, MRPS34, MRPL17, YARS2, MRPL1, SHMT2, TFB1M, LARS2, SUPV3L1, TFAM, CDK5RAP1, TUFM, MRPS18A, MTERF3, RPUSD4, C1QBP, EARS2, GFM1, MRPS15, AARS2, MRPL22, MRPL37, MRPS18B, MRPL20, DARS2, CHCHD1, MRPL13, RARS2, MRPL19, MRPL48, MRPS6, SLC25A33, MRPL3, MRPS31, FASTKD5, MRPL11, TRUB2, MRPL28, TSFM, MRPL58, GFM2, PTCD3, MRPL30, MRPL2, QRSL1, ALKBH1, MRPL10, MPV17L2
RNA splicing, via transesterification reactions	-2.25	7.54e-9	193	330	CASC3, SNU13, U2AF2, PNN, SNRPA1, CELF3, HNRNPA1, CELF4, WBP4, CWC22, SF3B5, RNVU1-3, RBM7, GEMIN8, NSRP1, WDR77, SRSF6, SART1, DDX20, PRPF40B, SETX, SRSF4, RBM8A, TXNL4A, RBM6, RBM15B, RBM42, SNRPE, TFIP11, CDC40, LUC7L3, PRPF19, SNRPB, SNIP1, GEMIN7, SF3B6, HNRNPH1, RNU6ATAC, RBM44, RBM23, PTBP1, SNRPC, DDX5, SNRPF, RBFOX2, ESS2, RBM10, USP49, SF3B1, GPATCH1, RALY, YJU2, WTAP, ZCCHC8, PRMT7, RNPS1, FRG1, RBMX2, MTREX, LSM4, MAGOHB, PLRG1, SF3A1, HNRNPH3, PPIE, DDX23, PUF60, DCPS, SRRM2, SF3B3, SNRPB2, PRPF18, GEMIN5, PRPF6, RBM17, SMU1, PSIP1, DNAJC17, EFTUD2, SNRPD2, SRSF2, NCL, USP4, SRSF7, SNRPD1, KHDRBS1, GEMIN4, YTHDC1, RBM3, METTL14, HNRNPR, FMR1, THRAP3, RBM15, ISY1, LSM5, IK, RSRC1, LARP7, HNRNPL, DHX8, TRA2A, BUD13, RBPMS, SNRPD3, LUC7L, SF3A3, SF3A2, PRPF8, HNRNPA3, SNW1, DDX41, STRAP, EIF4A3, SON, DDX1, PHF5A, CDC5L, UPF3B, PRPF40A, HNRNPF, NUP98, MAGOH, TIA1, KHSRP, C1QBP, PRMT5, PPIL1, HNRNPU, ZMAT2, HNRNPM, HNRNPC, HNRNPA2B1, DHX9, PPWD1, USP39, GEMIN6, PRPF31, DAZAP1, HNRNPK, SYNCRIP, HNRNPUL2, SFSWAP, CTNNBL1, AQR, SNRPA, HNRNPUL1, SNRNP25, CWC27, DYRK1A, HSPA8, RBMX, SNRNP200, SNRNP40, BCAS2, RBM14, AAR2, METTL16, PRPF4, SRPK1, RBM39, DHX15, SRSF3, SF1, SART3, NPM1, DHX35, SNRPG, COIL, CWF19L2, ZCRB1, NOVA2, DDX46, EXOSC10, NCBP1, MFAP1, LSM3, SRSF1, SRRM1, TRA2B, LSM2, SFPQ, PPIH, XAB2, GEMIN2, PTBP2, CRNKL1, MPHOSPH10, CWC15, PRPF38A, CWF19L1, DDX39A, SF3B2
RNA splicing, via transesterification reactions with bulged adenosine as nucleophile	-2.25	7.54e-9	191	326	CASC3, SNU13, U2AF2, PNN, SNRPA1, CELF3, HNRNPA1, CELF4, WBP4, CWC22, SF3B5, RNVU1-3, RBM7, GEMIN8, NSRP1, WDR77, SRSF6, SART1, DDX20, PRPF40B, SETX, SRSF4, RBM8A, TXNL4A, RBM6, RBM15B, RBM42, SNRPE, TFIP11, CDC40, LUC7L3, PRPF19, SNRPB, SNIP1, GEMIN7, SF3B6, HNRNPH1, RNU6ATAC, RBM44, RBM23, PTBP1, SNRPC, DDX5, SNRPF, RBFOX2, ESS2, RBM10, USP49, SF3B1, GPATCH1, RALY, YJU2, WTAP, ZCCHC8, PRMT7, RNPS1, FRG1, RBMX2, MTREX, LSM4, MAGOHB, PLRG1, SF3A1, HNRNPH3, PPIE, DDX23, PUF60, DCPS, SRRM2, SF3B3, SNRPB2, PRPF18, GEMIN5, PRPF6, RBM17, SMU1, PSIP1, DNAJC17, EFTUD2, SNRPD2, SRSF2, NCL, USP4, SRSF7, SNRPD1, KHDRBS1, GEMIN4, YTHDC1, RBM3, METTL14, HNRNPR, FMR1, THRAP3, RBM15, ISY1, LSM5, IK, RSRC1, LARP7, HNRNPL, DHX8, TRA2A, BUD13, RBPMS, SNRPD3, LUC7L, SF3A3, SF3A2, PRPF8, HNRNPA3, SNW1, DDX41, STRAP, EIF4A3, SON, DDX1, PHF5A, CDC5L, UPF3B, PRPF40A, HNRNPF, NUP98, MAGOH, TIA1, C1QBP, PRMT5, PPIL1, HNRNPU, ZMAT2, HNRNPM, HNRNPC, HNRNPA2B1, DHX9, PPWD1, USP39, GEMIN6, PRPF31, DAZAP1, HNRNPK, SYNCRIP, HNRNPUL2, SFSWAP, CTNNBL1, AQR, SNRPA, HNRNPUL1, SNRNP25, CWC27, DYRK1A, HSPA8, RBMX, SNRNP200, SNRNP40, BCAS2, RBM14, AAR2, METTL16, PRPF4, SRPK1, RBM39, DHX15, SRSF3, SF1, SART3, NPM1, DHX35, SNRPG, COIL, CWF19L2, ZCRB1, NOVA2, DDX46, EXOSC10, NCBP1, MFAP1, LSM3, SRSF1, SRRM1, TRA2B, LSM2, SFPQ, PPIH, XAB2, GEMIN2, PTBP2, CRNKL1, CWC15, PRPF38A, CWF19L1, DDX39A, SF3B2
mRNA splicing, via spliceosome	-2.25	7.54e-9	191	326	CASC3, SNU13, U2AF2, PNN, SNRPA1, CELF3, HNRNPA1, CELF4, WBP4, CWC22, SF3B5, RNVU1-3, RBM7, GEMIN8, NSRP1, WDR77, SRSF6, SART1, DDX20, PRPF40B, SETX, SRSF4, RBM8A, TXNL4A, RBM6, RBM15B, RBM42, SNRPE, TFIP11, CDC40, LUC7L3, PRPF19, SNRPB, SNIP1, GEMIN7, SF3B6, HNRNPH1, RNU6ATAC, RBM44, RBM23, PTBP1, SNRPC, DDX5, SNRPF, RBFOX2, ESS2, RBM10, USP49, SF3B1, GPATCH1, RALY, YJU2, WTAP, ZCCHC8, PRMT7, RNPS1, FRG1, RBMX2, MTREX, LSM4, MAGOHB, PLRG1, SF3A1, HNRNPH3, PPIE, DDX23, PUF60, DCPS, SRRM2, SF3B3, SNRPB2, PRPF18, GEMIN5, PRPF6, RBM17, SMU1, PSIP1, DNAJC17, EFTUD2, SNRPD2, SRSF2, NCL, USP4, SRSF7, SNRPD1, KHDRBS1, GEMIN4, YTHDC1, RBM3, METTL14, HNRNPR, FMR1, THRAP3, RBM15, ISY1, LSM5, IK, RSRC1, LARP7, HNRNPL, DHX8, TRA2A, BUD13, RBPMS, SNRPD3, LUC7L, SF3A3, SF3A2, PRPF8, HNRNPA3, SNW1, DDX41, STRAP, EIF4A3, SON, DDX1, PHF5A, CDC5L, UPF3B, PRPF40A, HNRNPF, NUP98, MAGOH, TIA1, C1QBP, PRMT5, PPIL1, HNRNPU, ZMAT2, HNRNPM, HNRNPC, HNRNPA2B1, DHX9, PPWD1, USP39, GEMIN6, PRPF31, DAZAP1, HNRNPK, SYNCRIP, HNRNPUL2, SFSWAP, CTNNBL1, AQR, SNRPA, HNRNPUL1, SNRNP25, CWC27, DYRK1A, HSPA8, RBMX, SNRNP200, SNRNP40, BCAS2, RBM14, AAR2, METTL16, PRPF4, SRPK1, RBM39, DHX15, SRSF3, SF1, SART3, NPM1, DHX35, SNRPG, COIL, CWF19L2, ZCRB1, NOVA2, DDX46, EXOSC10, NCBP1, MFAP1, LSM3, SRSF1, SRRM1, TRA2B, LSM2, SFPQ, PPIH, XAB2, GEMIN2, PTBP2, CRNKL1, CWC15, PRPF38A, CWF19L1, DDX39A, SF3B2
Mitochondrial translation	-2.23	7.54e-9	93	128	MRPL54, IARS2, MRPS5, MRPL43, MALSU1, MRPS28, MTRF1L, MRPS11, MRPL12, MTIF3, MRPS16, MRPL21, PTCD1, MRPL55, GADD45GIP1, MRPL34, MRPS35, MTIF2, MRPL9, MRPL50, MRPS18C, MRPL42, RMND1, UQCC2, GATC, TACO1, MRPL40, MRPL32, MRPL16, MRPL44, MTG2, MRPS14, MRPL15, FASTKD2, RCC1L, MRPL45, MRPS17, MRPS26, MRPS7, TARS2, MRPL35, WARS2, TRMT10C, MRPS27, MRPS22, LRPPRC, FASTKD3, MRPL47, MRPS9, MRPL23, MRPS2, MRPL4, MRPS34, MRPL17, YARS2, MRPL1, SHMT2, LARS2, CDK5RAP1, TUFM, MRPS18A, RPUSD4, C1QBP, EARS2, GFM1, MRPS15, AARS2, MRPL22, MRPL37, MRPS18B, MRPL20, DARS2, CHCHD1, MRPL13, RARS2, MRPL19, MRPL48, MRPS6, MRPL3, MRPS31, MRPL11, TRUB2, MRPL28, TSFM, MRPL58, GFM2, PTCD3, MRPL30, MRPL2, QRSL1, ALKBH1, MRPL10, MPV17L2
Mitotic sister chromatid segregation	-2.22	7.54e-9	129	188	PINX1, MZT1, KIF4B, UBE2C, XRCC3, ANKRD53, KAT5, CCSAP, PRC1, DCTN2, PIBF1, CDC16, LCMT1, PINX1, PSRC1, RANGRF, ATM, UHRF1, ANAPC7, APC, BUB3, STAG1, ANAPC11, CDK1, MAP10, KIF2A, SPAG5, KIF22, NSL1, NCAPD2, KIF18B, KIF23, MAD1L1, SMC1A, KNTC1, NDC80, RACGAP1, ESPL1, SPDL1, CHMP2A, ANAPC5, NUF2, KMT5A, AAAS, DRG1, SMC4, ZNF207, CDT1, CENPC, TUBG1, INCENP, NCAPH2, CCNB1, CDCA5, BIRC5, TPX2, KNSTRN, KIF14, NUDC, CENPF, KIF11, KLHL22, IK, NUSAP1, PPP2R1A, NCAPG, WAPL, SMARCA5, CENPK, ZW10, BAZ1B, MAD2L1, KIF4A, TEX14, LSM14A, KIF18A, ZWINT, PHF13, NCAPG2, HNRNPU, NUP62, KIF2C, CENPE, SEH1L, TTK, DLGAP5, HASPIN, AURKB, TRIP13, KPNB1, CDCA8, SPC25, KIFC1, BCCIP, INO80, FBXO5, SMC2, RAN, SMC3, PSMG2, NIPBL, KNL1, RHOA, PLK1, MAPRE1, CENPI, BUB1B, SKA3, ABRAXAS1, NCAPD3, GEN1, CHAMP1, CDK5RAP2, CDC20, SKA1, DYNC1LI1, ZWILCH, RAD21, BUB1, DIS3L2, CCDC61, CEP192, MYBL2, SKA2, NCAPH, MIS12, ABRAXAS2, BOD1, CDC23
Regulation of chromosome segregation	-2.21	7.54e-9	92	128	UBE2C, XRCC3, KAT5, RCC2, CDC16, LCMT1, ATM, ANAPC7, APC, BUB3, ANAPC11, CDK1, BCL7B, ARID1B, NCAPD2, TACC3, MAD1L1, KNTC1, NDC80, ESPL1, SPDL1, ANAPC5, NUF2, PBRM1, SMC4, ZNF207, PHF10, CSNK2A2, INCENP, NCAPH2, CCNB1, BIRC5, CENPF, SMC6, CDC27, CDC6, PUM1, KLHL22, IK, ACTB, NCAPG, ZW10, ARID2, MAD2L1, RIOK2, TEX14, ZWINT, PUM2, DPF2, ANAPC1, NCAPG2, HNRNPU, BCL7A, SMARCD3, KIF2C, CENPE, TTK, RMI2, DLGAP5, HASPIN, MKI67, AURKB, TRIP13, SMARCA4, CDCA8, SPC25, FBXO5, SMC2, PSMG2, KNL1, PLK1, CDCA2, ANAPC4, BUB1B, SMARCD1, BRD7, NCAPD3, GEN1, SMARCC1, NEK6, CDK5RAP2, CDC20, DYNC1LI1, ZWILCH, SMC5, RAD21, BUB1, CSNK2A1, ACTL6A, NCAPH, DPF1, CDC23
Sister chromatid segregation	-2.21	7.54e-9	155	229	PINX1, MZT1, KIF4B, UBE2C, XRCC3, ANKRD53, KAT5, CCSAP, PRC1, DCTN2, PIBF1, CDC16, LCMT1, PINX1, PSRC1, RANGRF, ATM, UHRF1, ANAPC7, APC, BUB3, STAG1, ANAPC11, CDK1, MAP10, KIF2A, SPAG5, BCL7B, ARID1B, KIF22, NSL1, NCAPD2, KIF18B, TACC3, KIF23, MAD1L1, SMC1A, KNTC1, NDC80, RACGAP1, ESPL1, SPDL1, CHMP2A, ANAPC5, NUF2, KMT5A, PBRM1, AAAS, TOP2A, DRG1, SMC4, ZNF207, CDT1, CENPC, TUBG1, LATS1, PHF10, INCENP, NCAPH2, CCNB1, CDCA5, BIRC5, TPX2, KNSTRN, KIF14, NUDC, CENPF, CDC27, CDC6, KIF11, TOP2B, KLHL22, IK, NUSAP1, ACTB, PPP2R1A, NCAPG, WAPL, SMARCA5, CENPK, ZW10, ARID2, BAZ1B, MAD2L1, RIOK2, KIF4A, TEX14, LSM14A, KIF18A, ZWINT, PHF13, DPF2, ANAPC1, NCAPG2, HNRNPU, NUP62, BCL7A, SMARCD3, KIF2C, CENPE, SEH1L, TTK, RMI2, DLGAP5, HASPIN, AURKB, TRIP13, SMARCA4, KPNB1, CDCA8, SPC25, KIFC1, BCCIP, INO80, FBXO5, SMC2, RAN, SMC3, PSMG2, NIPBL, KNL1, RHOA, PLK1, MAPRE1, ANAPC4, CENPI, BUB1B, SKA3, SMARCD1, BRD7, ABRAXAS1, NCAPD3, GEN1, SMARCC1, CHAMP1, NEK6, CDK5RAP2, CDC20, SKA1, DYNC1LI1, ZWILCH, RAD21, BUB1, DIS3L2, CCDC61, CEP192, MYBL2, SKA2, ACTL6A, NCAPH, MIS12, ABRAXAS2, DPF1, BOD1, CDC23
RNA 3'-end processing	-2.21	7.54e-9	73	97	PARN, RPS21, TRNT1, RPRD1B, PTCD1, SLBP, ZC3H3, POLR2D, LSM11, PCF11, CSTF1, INTS6, RPRD2, INTS2, SSB, PNPT1, APP, ZC3H14, MTPAP, INTS14, LEO1, INTS8, INTS5, PAF1, INTS7, TRMT10C, ELAC2, ERI2, EXOSC2, HSD17B10, EXOSC5, TUT4, INTS1, RPRD1A, EXOSC8, FBL, INTS12, SCAF8, DHX36, YTHDC1, LARP7, ZNF473, SUPV3L1, ELAC1, EXOSC7, BARD1, EXOSC3, SYMPK, EXOSC9, TOE1, TUT1, DKC1, LIN28B, CPSF7, NUDT21, CPSF6, ERI3, LIN28A, INTS9, CSTF3, AHCYL1, EXOSC10, NCBP1, CSTF2, SSU72, WDR33, CSTF2T, CPSF3, GRSF1, PAPOLA, ERI1, CPSF2, VIRMA
Regulation of chromosome organization	-2.21	7.54e-9	167	244	ZNF304, ACTR8, NFRKB, NSMCE3, INO80E, SMARCD2, SMG1, ACD, NBN, SPC24, PRAP1, ATRX, SMARCC2, SETDB2, PINX1, PARN, UBE2C, SENP6, XRCC3, HMBOX1, KAT5, HNRNPA1, CDC16, LCMT1, ATR, PINX1, ATM, GTF2H2, MAPK1, ANAPC7, APC, UCHL5, YY1, BUB3, CCT3, ANAPC11, TRAPPC12, CDK1, MCPH1, PARP1, MCRS1, XRN1, PPHLN1, BCL7B, NAF1, RUVBL1, CCT2, USP7, RUVBL2, CCT5, ARID1B, NCAPD2, TP53, TACC3, MAD1L1, KNTC1, NDC80, ESPL1, NSMCE1, ACTR5, SPDL1, ANAPC5, NUF2, PBRM1, TOP2A, RNF4, NAT10, SMC4, ZNF207, PHF10, INCENP, NCAPH2, CCNB1, BIRC5, DHX36, ATF7IP, CENPF, FEN1, POT1, SMC6, DCP2, CDC27, CDC6, KLHL22, IK, ACTB, NCAPG, WAPL, SMARCA5, ZW10, ARID2, MPHOSPH8, XRCC1, BAZ1B, NEK7, MAD2L1, RIOK2, CCT4, DDX11, TEX14, HNRNPD, ZWINT, CCT7, DPF2, ANAPC1, NCAPG2, HNRNPU, BCL7A, SMARCD3, HNRNPC, HNRNPA2B1, SETMAR, CTNNB1, CENPE, TTK, DKC1, TNKS, CCT8, RMI2, TERF1, ERCC4, CCT6A, DLGAP5, XRCC5, SLF2, HASPIN, TRIM28, TCP1, AURKB, TRIP13, SMARCA4, CDCA8, SPC25, CTCF, INO80, FBXO5, SMC2, PSMG2, KNL1, MRE11, PLK1, ANAPC4, BUB1B, SMARCD1, BRD7, NCAPD3, SUB1, GEN1, SMARCC1, NEK6, CDK5RAP2, CDC20, DYNC1LI1, ZWILCH, SMC5, RAD21, BUB1, EXOSC10, ACTL6A, FBXO4, NCAPH, NABP2, ATF7IP2, SLF1, DPF1, MAP3K4, CDC23

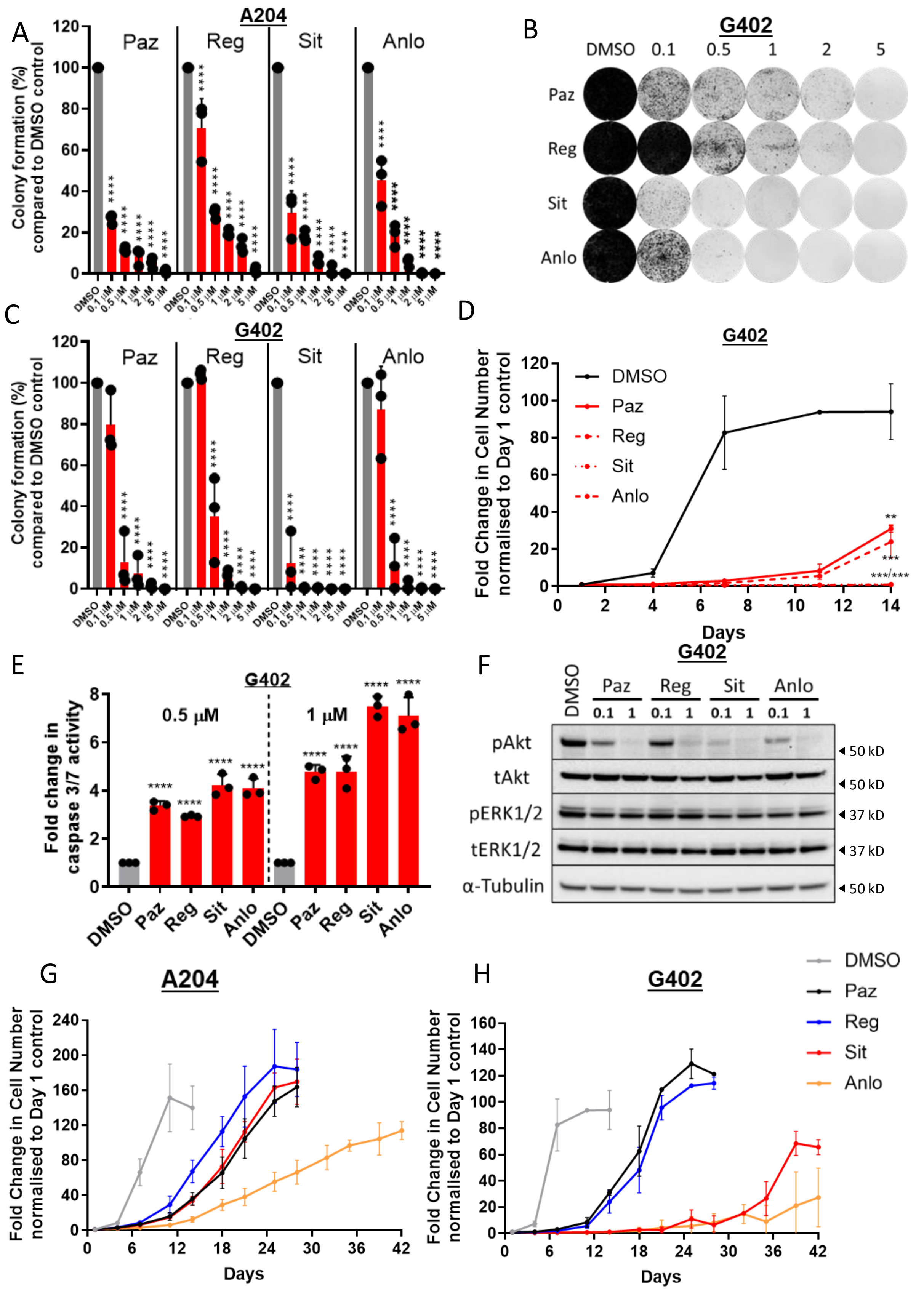

Supplemental Figure 1

Figure S1. Malignant rhabdoid tumour cells are sensitive to pazopanib, regorafenib, sitravatinib, and anlotinib. (A) Quantification of A204 colony formation assays normalised to DMSO control. Statistical analysis was undertaken using one-way ANOVA with Dunnett's multiple comparison tests (DMSO control). Error bars represent

standard deviation (n=3). **(B)** Colony formation assays of G402 cells treated with increasing concentrations of mTKIs over a period of 2 weeks. Image is representative of three separate experiments. **(C)** Quantification of G402 colony formation assays which was normalised to DMSO control. Statistical analysis was undertaken using one-way ANOVA with Dunnett's multiple comparison tests (DMSO control). Error bars represent standard deviation. **(D)** Growth curve assays of G402 to measure the fold change in cell number over a period of 2 weeks. Fold change was normalised to day 1 control (n=2). Error bars represent the standard deviation. Statistical analysis was undertaken using one-way ANOVAs with Dunnett's multiple comparison tests (DMSO control). **(E)** Bar plots displaying the fold change in caspase 3/7 activity in the G402 cells treated with two concentrations of mTKIs for 24 hours. Fold change was normalised to DMSO control (n=3). Error bars represent standard deviation. Statistical analysis was undertaken using one-way ANOVA with Dunnett's multiple comparison tests (DMSO control) (* = p ≤ 0.05, ** = p ≤ 0.01, *** = p ≤ 0.001, **** = p ≤ 0.0001). **(F)** Immunoblot of Akt and ERK1/2 signalling modulation in G402 cells after 6 hours of treatment with either 0.1 or 1 μM of mTKIs. Image is representative of two separate experiments. Growth curve assays of A204 **(G)** or G402 **(H)** to measure the fold change in cell number over a period of 6 weeks. Fold change was normalised to day 1 control (n=4 for A204; n=2 for G402). Error bars represent the standard deviation. Paz: Pazopanib, Reg: Regorafenib, Sit: Sitravatinib and Anlo: Anlotinib.

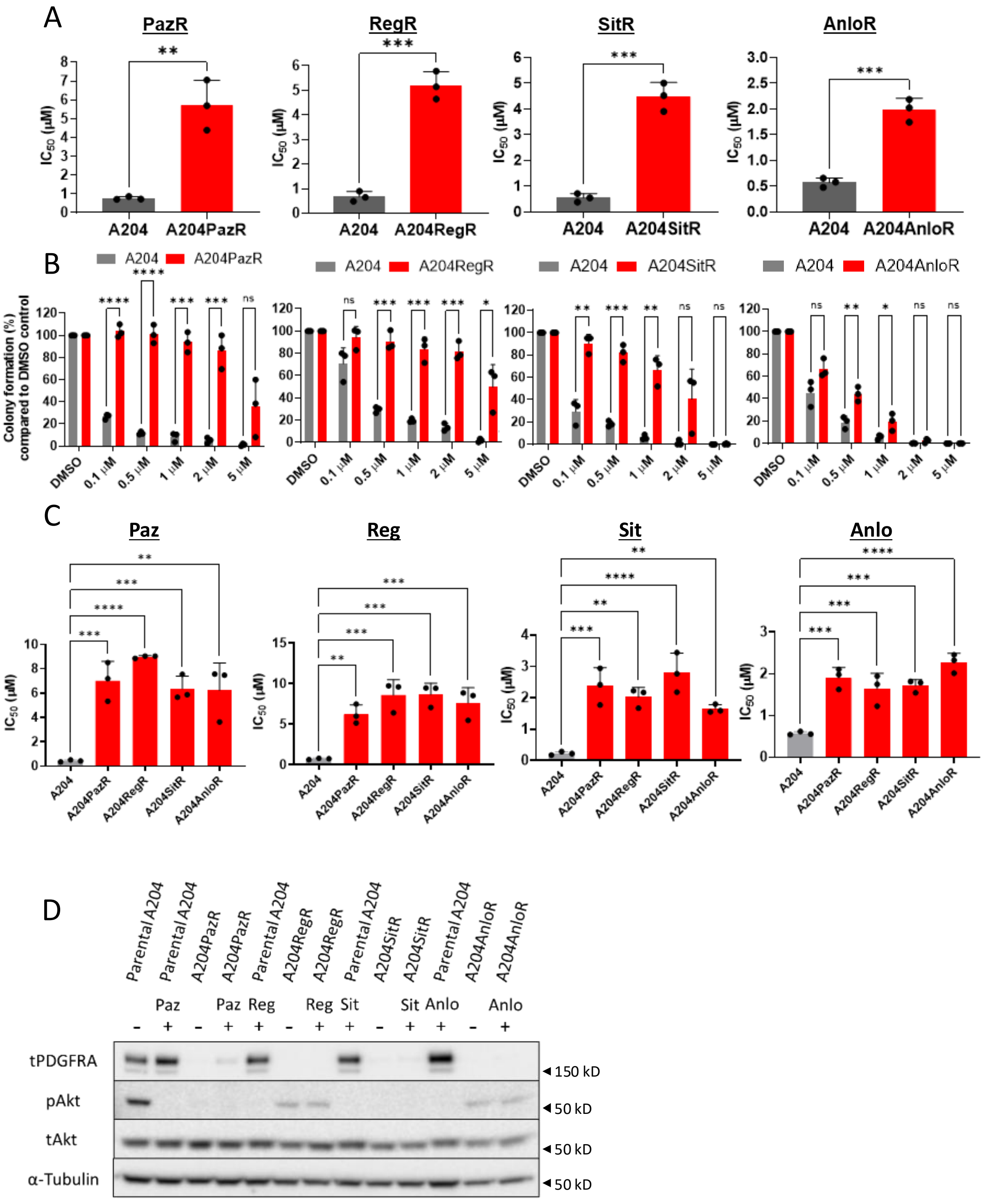

Supplemental Figure 2

Figure S2. Characterisation of A204 mTKI-resistant sublines. (A) Bar plots displaying IC_{50} values for A204 parental and mTKI-resistant sublines treated with their respective mTKI for 72h hours. Cell viability was normalised to DMSO control. Error bars represent standard deviation (n=3). Statistical analysis was undertaken by Student's unpaired T tests. **(B)** Quantification of A204 parental and mTKI-resistant subline colony formation assays normalised to DMSO control. Statistical analysis was undertaken by Student's unpaired T tests. Error bars represent standard deviation (n=3). **(C)** Bar plots displaying IC_{50} values for A204 parental and mTKI-resistant sublines treated with mTKIs for 72 hours. Cell viability was normalised to DMSO control. Error bars represent standard deviation (n=3). Statistical analysis was undertaken by one-way ANOVA with Dunnett's multiple comparison tests (parental A204 control) (* = $p \leq 0.05$, ** = $p \leq 0.01$, *** = $p \leq 0.001$, **** = $p \leq 0.0001$). **(D)**

Immunoblot of PDGFRA and Akt expression and/or phosphorylation levels after 6 hours of treatment with 1 μM of indicated mTKI. Image is representative of two separate experiments (n=2). Paz: Pazopanib, Reg: Regorafenib, Sit: Sitravatinib, and Anlo: Anlotinib.

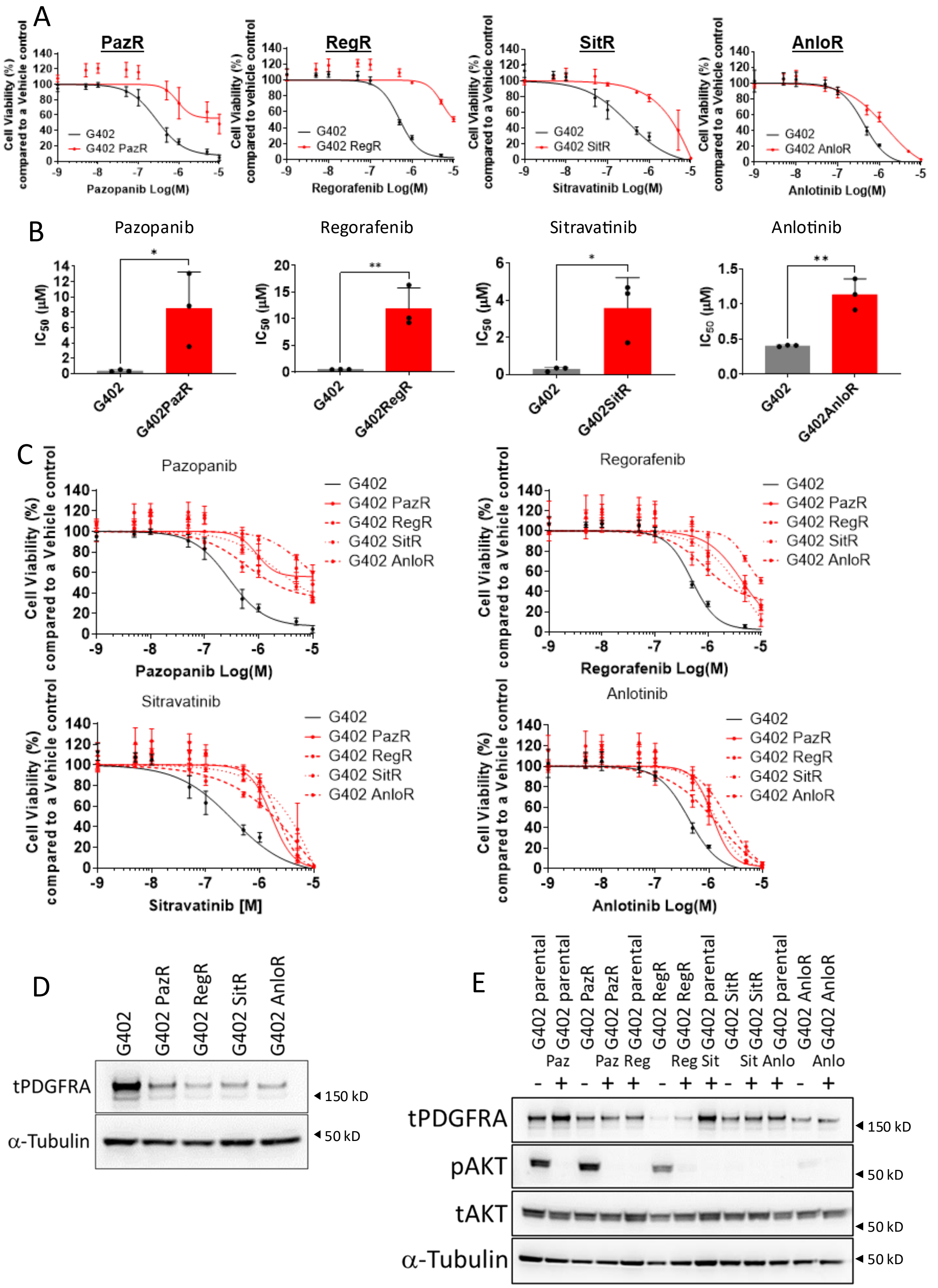

Supplemental Figure 3

Figure S3. Characterisation of G402 mTKI-resistant sublines. (A) Dose response assays of G402PazR, G402RegR, G402SitR, and G402AnloR cells treated with increasing concentrations (treated for 72 hours) with their respective inhibitor to determine IC_{50} values. Cell viability was normalised to DMSO control. Error bars represent standard deviation (n=3). **(B)** Bar plots displaying IC_{50} values for G402 parental and mTKI-resistant sublines treated

with their respective mTKI. Cell viability was normalised to DMSO control. Error bars represent standard deviation (n=3). Statistical analysis was undertaken by Student's unpaired T tests (* = $p \leq 0.05$, ** = $p \leq 0.01$). **(C)** Dose response assays of G402 mTKI-resistant sublines treated with increasing concentrations (treated for 72 hours) of mTKIs to determine IC_{50} values. Cell viability was normalised to DMSO control. Error bars represent standard deviation (n=3). **(D)** Immunoblot of baseline PDGFRA expression levels in G402 parental and TKI-resistant sublines. Image is representative of two separate experiments. **(E)** Immunoblot of G402 PDGFRA and Akt expression and/or phosphorylation levels after 6 hours of treatment with 1 µM of indicated mTKI. Image is representative of two separate experiments (n=2). Paz: Pazopanib, Reg: Regorafenib, Sit: Sitravatinib, and Anlo: Anlotinib.

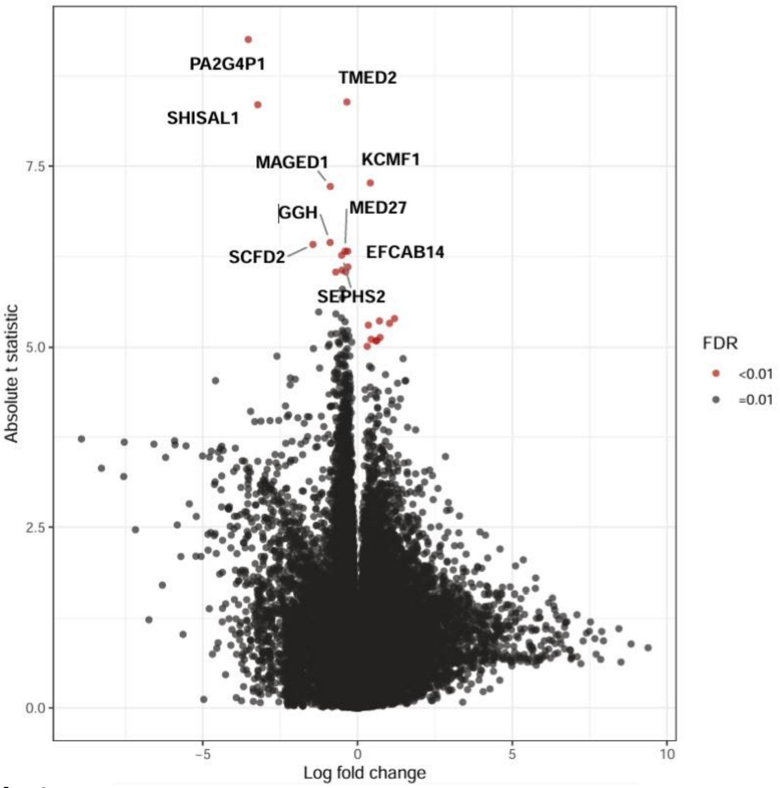

Supplemental Figure 4

Figure S4. Volcano plot of differentially expressed genes in A204 and G402 drug-resistant versus parental cell lines. Multiple testing correction was performed using Benjamini-Hochberg at 1% false discovery rate (FDR). Labels indicate the top 5 up- and down-regulated genes by absolute t-statistic.

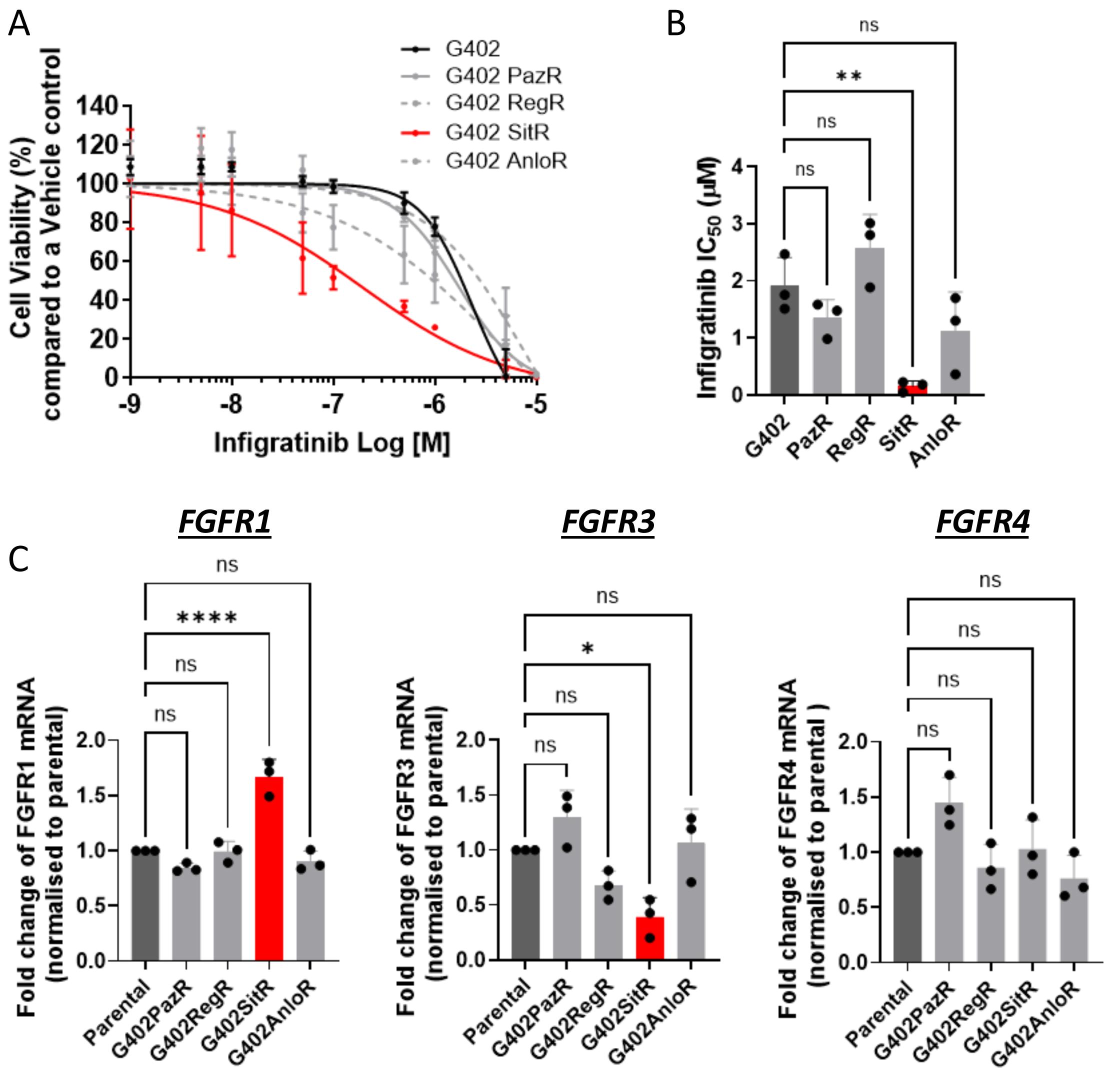

Supplemental Figure 5

Figure S5. Collateral sensitivity assessment in G402 mTKI-resistant sublines. (A) Dose response assay of G402 parental and TKI-resistant sublines treated for 72 hours with increasing concentrations of infigratinib to determine IC_{50} values. Error bars represent standard deviation (n=3). **(B)** Bar plots displaying infigratinib IC_{50} values for A204 parental and mTKI-resistant sublines. Cell viability was normalised to DMSO control. Error bars represent standard deviation (n=3). Statistical analysis was undertaken by one-way ANOVAs with Dunnett's multiple comparison tests (parental G402) (** = $p \leq 0.01$). **(C)** qPCR data displaying the fold change of *FGFR1*, *FGFR3* and *FGFR4* mRNA, normalised to G402 parental cells (n=3). Statistical analysis was undertaken using one-way ANOVAs with Dunnett's multiple comparison tests (G402 parental) (* = $p \leq 0.05$ and ****$p \leq 0.0001$).

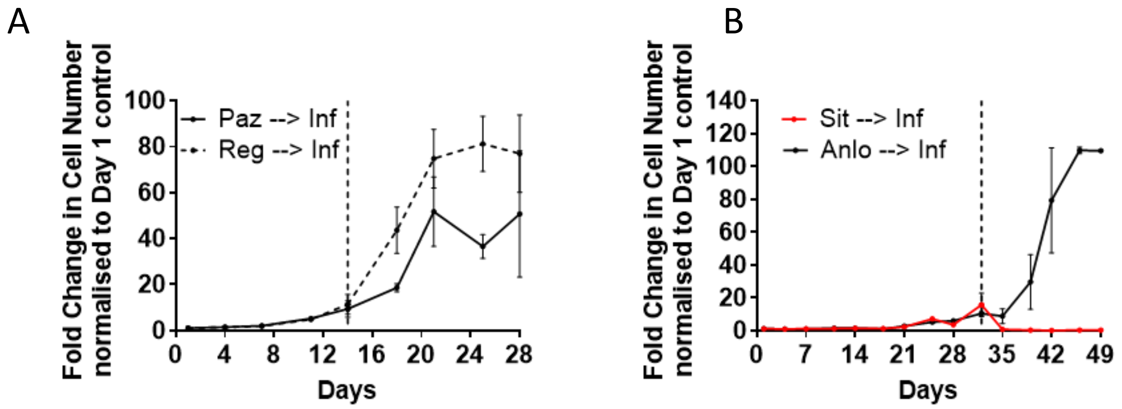

Supplemental Figure 6

Figure S6. Temporal assessment of infigratinib collateral sensitivity in G402 sitravatinib pre-treated cells. Growth curve assays of G402 cells to measure the fold change in cell number over a period of either 4 (left) or 7 weeks (right). iCells treated initially with 1 μM mTKIs for 2 weeks (Paz and Reg) or 4.5 weeks (Sit and Anlo), before switching to 1 μM infigratinib for a further 2 (Paz and Reg) or 2.5 weeks (Sit and Anlo). Fold change was normalised to day 1 control (n=2). Error bars represent standard deviation. Paz: Pazopanib, Reg: Regorafenib, Sit: Sitravatinib, Anlo: Anlotinib, and Inf: Infigratinib.

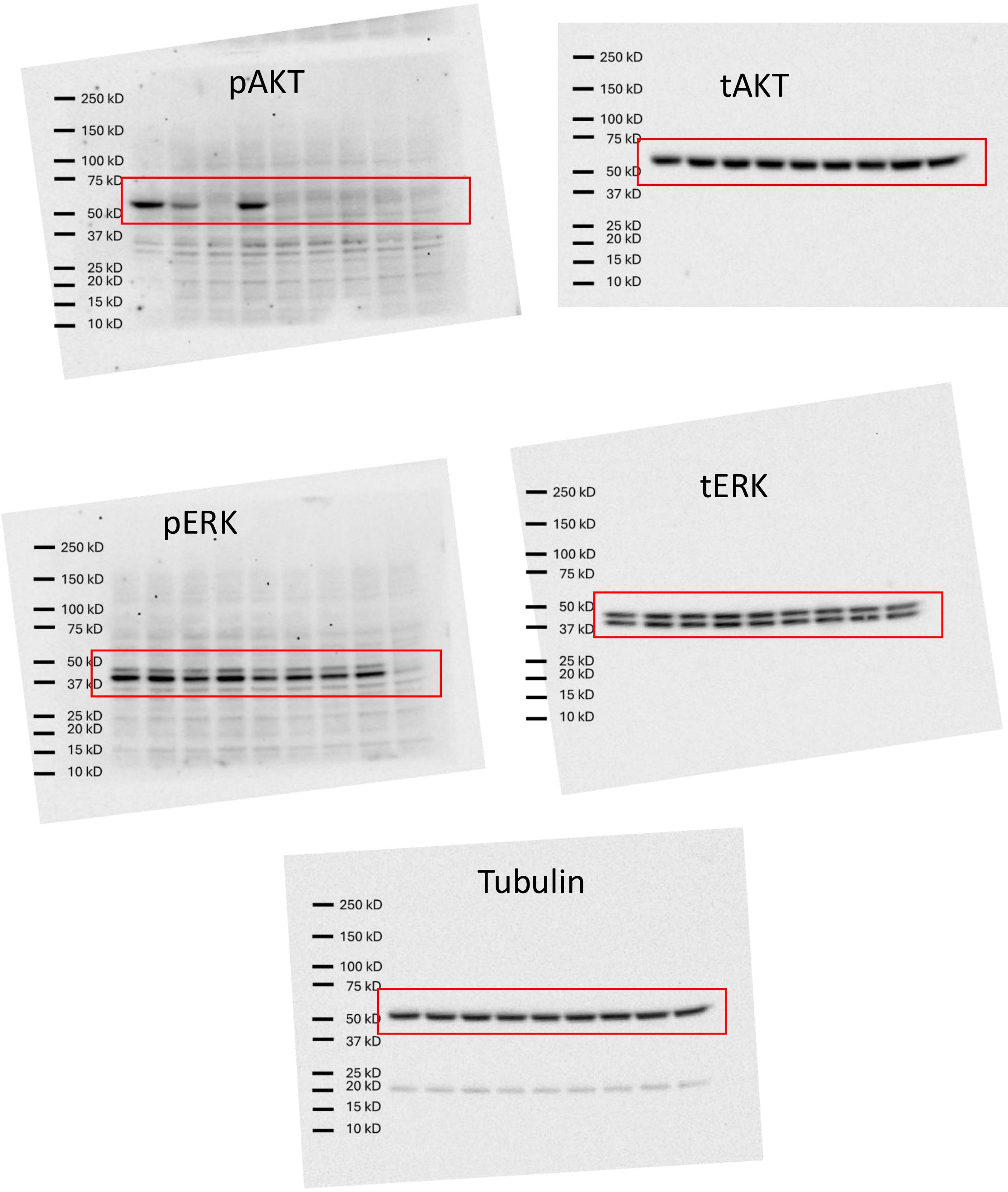

Supplemental Figure 7

Figure S7. Uncropped western blot images corresponding to Fig 1E. Bands used in the figure are highlighted in the red box.

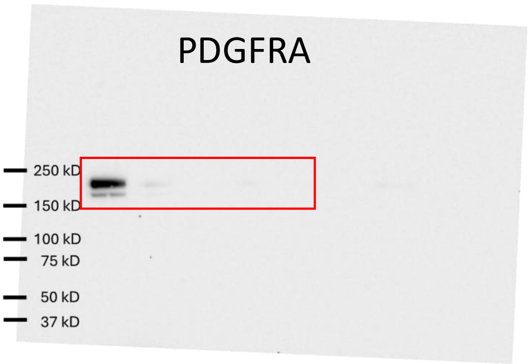

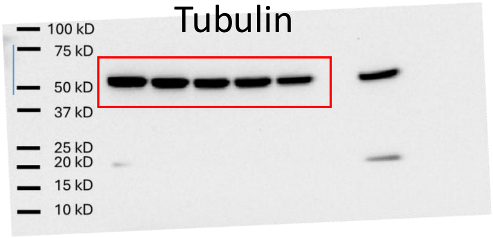

Supplemental Figure 8

Figure S8. Uncropped western blot images corresponding to Fig 2D. Bands used in the figure are highlighted in the red box.

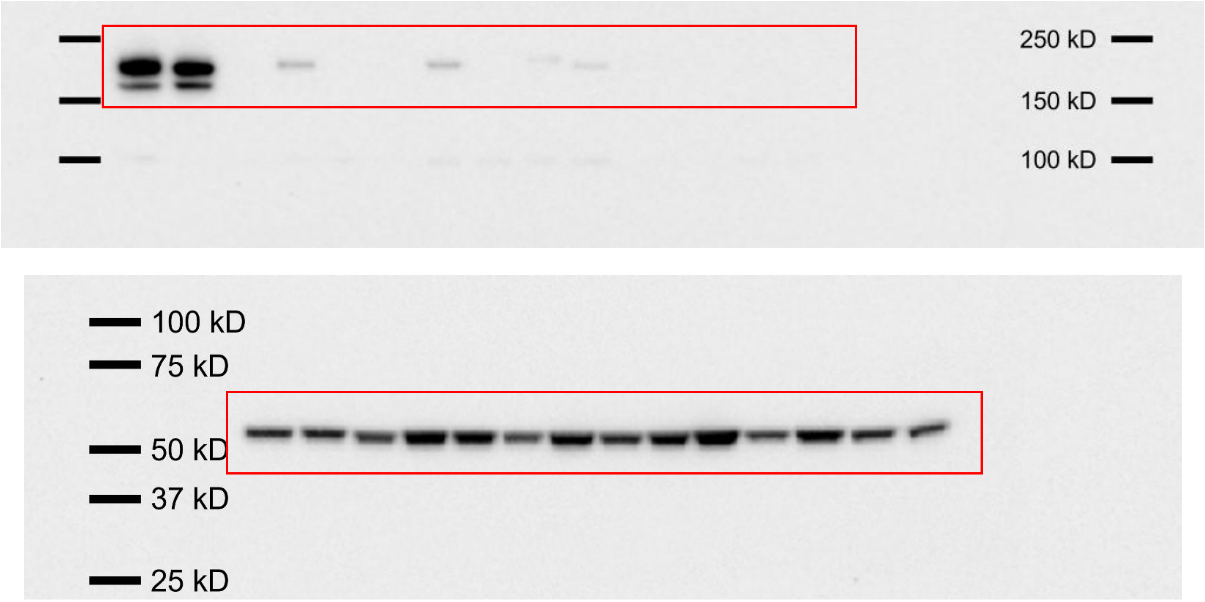

Supplemental Figure 9

Figure S9. Uncropped western blot images corresponding to Fig 3A. Bands used in the figure are highlighted in the red box.

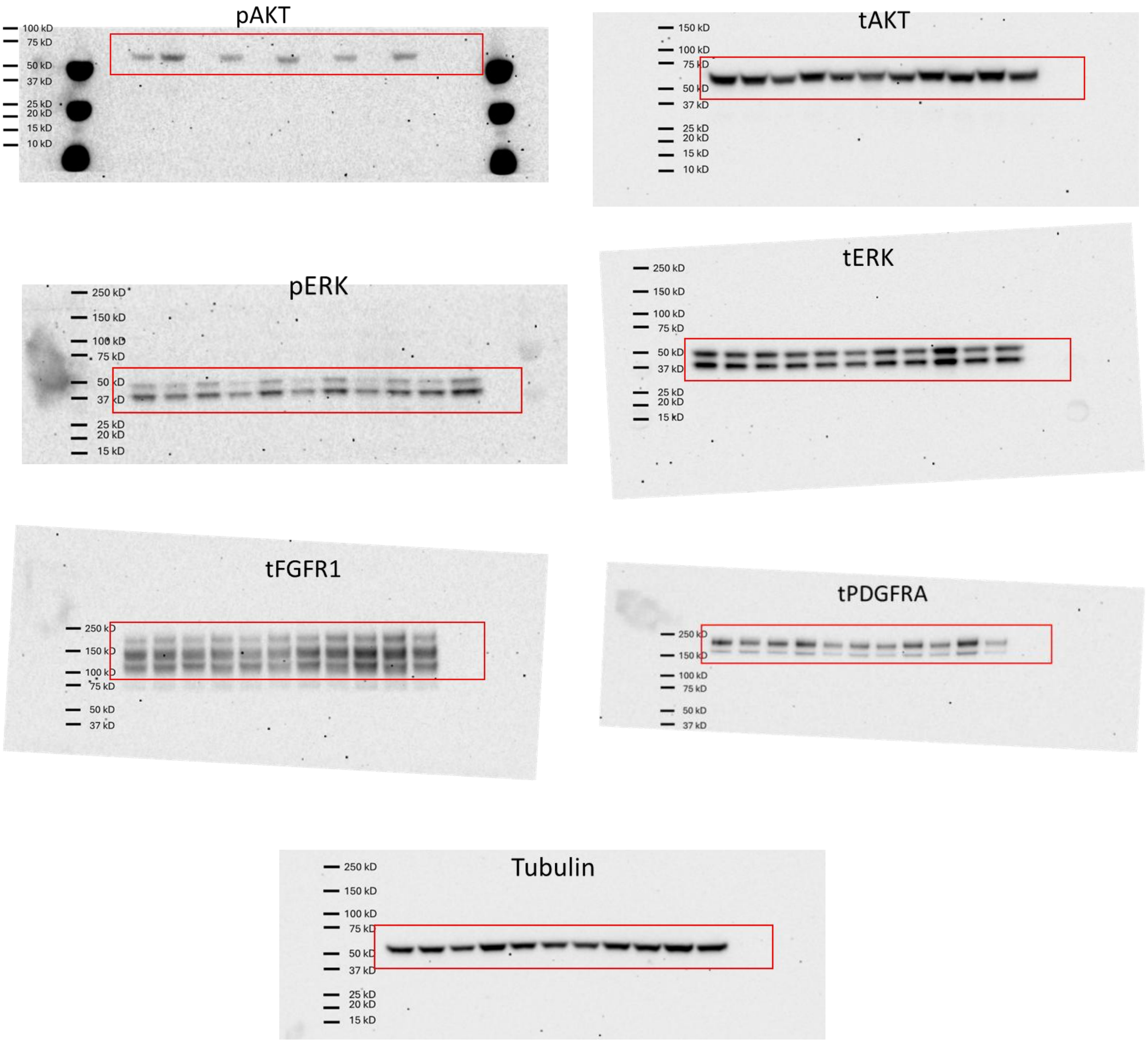

Supplemental Figure 10

Figure S10. Uncropped western blot images corresponding to Fig 7F. Bands used in the figure are highlighted in the red box.

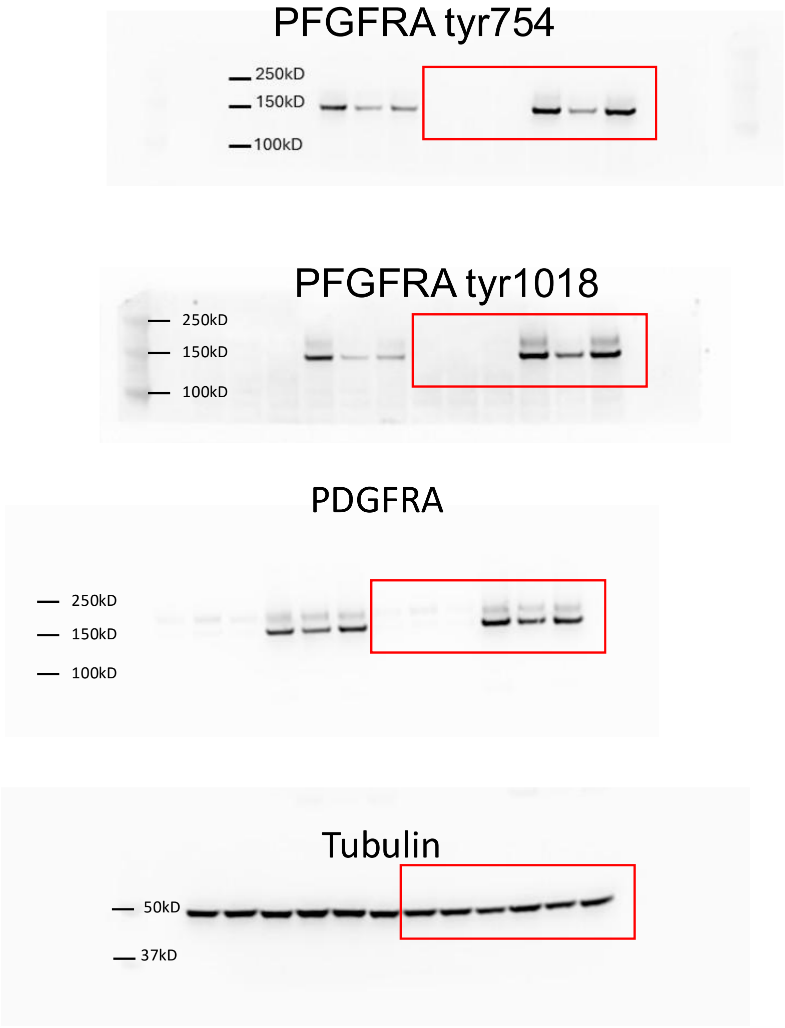

Supplemental Figure 11

Figure S11. Uncropped western blot images corresponding Fig 7H. Bands used in the figure are highlighted in the red box.

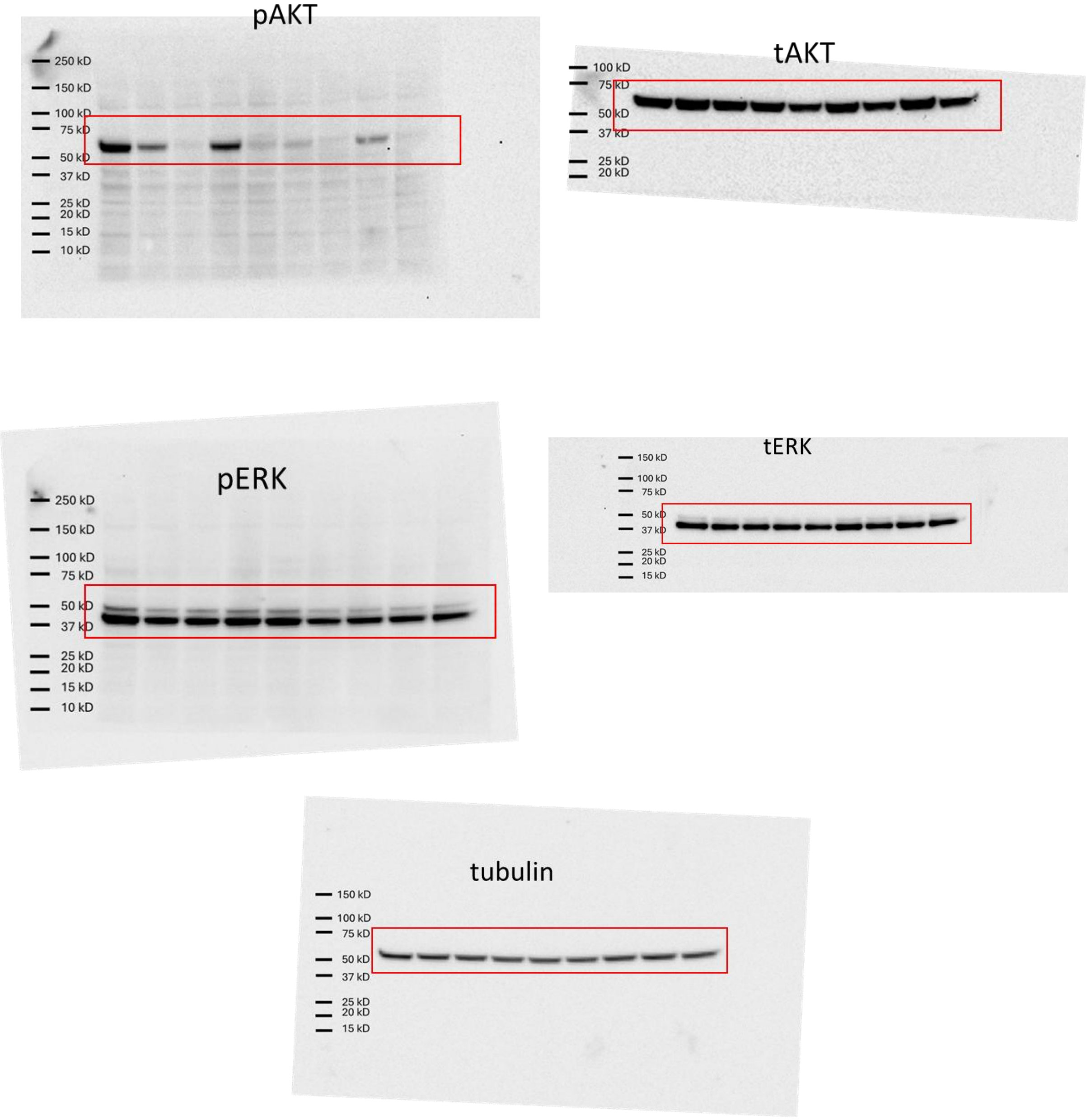

Supplemental Figure 12

Figure S12. Uncropped western blot images corresponding to Fig S1F. Bands used in the figure are highlighted in the red box.

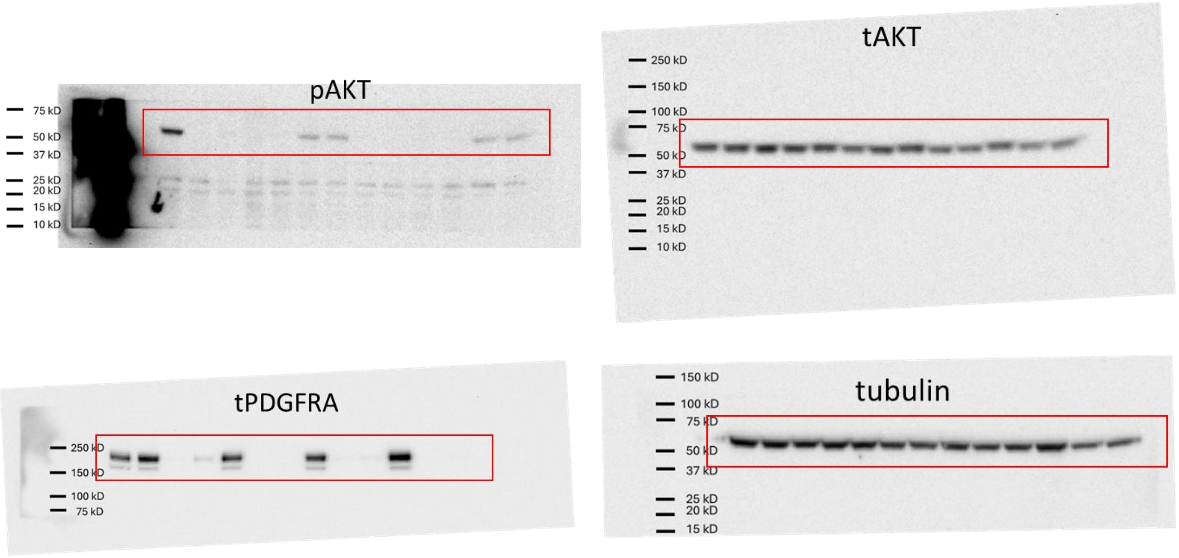

Supplemental Figure 13

Figure S13. Uncropped western blot images corresponding to Fig S2D. Bands used in the figure are highlighted in the red box.

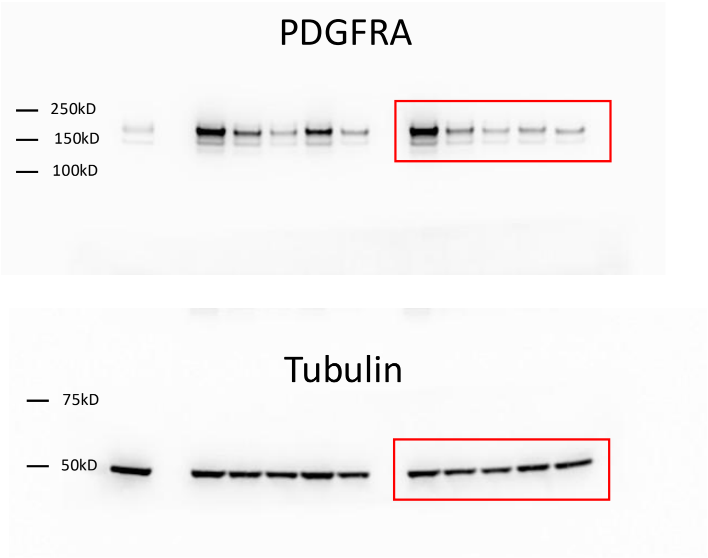

Supplemental Figure 14

Figure S14. Uncropped western blot images corresponding to Fig S3D. Bands used in the figure are highlighted in the red box.

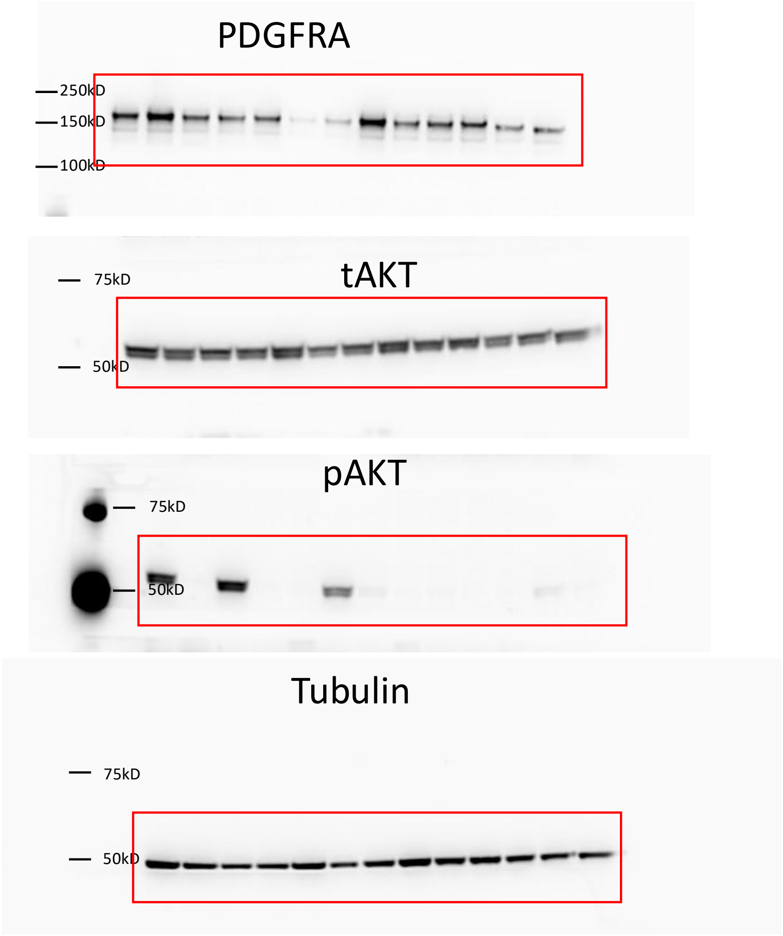

Supplemental Figure 15

Figure S15. Uncropped western blot images corresponding to Fig S3E. Bands used in the figure are highlighted in the red box.

Supplemental methods

Immunoblotting

Cells (350,000/well or 900,000/flask) were seeded into 6 well plates or T25 flasks. After 24 hours, cells were treated with inhibitors (or DMSO [Sigma Aldrich]) at the indicated concentrations and/or durations. At the indicated time period post treatment, cells were lysed in radioimmunoprecipitation assay (RIPA) buffer (50 mM Tris.Cl pH 7.6, 150 mM NaCl, 1% IGEPAL CA-640 [NP-40] [Sigma Aldrich], 0.1% sodium dodecyl sulfate [SDS] [Sigma Aldrich], and 0.5% sodium deoxycholate [Sigma Aldrich]), supplemented with Halt^{TM} protease and phosphatase inhibitors and EDTA #78442 (ThermoFisher Scientific) at 4 °C.

Lysates were loaded onto NuPAGE Novex 4-12% Bis-Tris gels (Invitrogen), followed by blotting onto iBlot PVDF membranes (Invitrogen). Membranes were blocked for 4-5 hours at room temperature with either 5% dried skimmed milk (Marvel) (for total protein) or 5% bovine serum albumin (BSA) (Sigma Aldrich) (for phosphoprotein) in 1x tris-buffered saline (TBS) with 0.1% Tween 20 (TBST) (Sigma Aldrich). Blots were subsequently incubated with primary antibodies (diluted in the respective blocking agent) at 4 °C overnight. Membranes were then washed at room temperature with 3 x 10 minutes of TBST incubation. Following this, membranes were incubated at room temperature with HRP-conjugated secondary antibodies (diluted in 5% dried skimmed milk) for 1 hour. Membranes were subsequently washed for a further 3 x 10 minutes of TBST incubation at room temperature. Immunoreactive bands were visualised by SuperSignal West Pico PLUS chemiluminescence substrate (ThermoFisher Scientific) and the blots were digitally imaged using ChemiDoc Touch Imaging System (Bio-Rad) or G-Box Chemi-XX6 (Syngene) imagers. Blots were analysed using Image Lab (Bio-Rad) and GNU image manipulation program (GIMP).

Phospho-RTK antibody array

Cells (1,000,000/flask) were seeded into T75 flasks. After 24 hours, T75s were treated with either DMSO (Sigma Aldrich) or 1 µM sitravatinib (Selleck Chemicals). Cells were allowed to grow for two weeks in their respective treatments. Media and inhibitors were replenished twice weekly. Cells were maintained at between 70-90% confluency and cells were split into T175s one-week post-seeding. After two weeks of treatment, the cells were treated for 6 hours with either DMSO, 1 µM infigratinib (Selleck Chemicals) or 1 µM sitravatinib. Cells were lysed and lysates collected and evaluated for phospho-RTK profiles using the Proteome Profiler Human Phospho-RTK Array kit (R & D Systems), as per the manufacturer's recommendations. RTK arrays were digitally imaged using the G-Box Chemi-XX6 (Syngene) imager. Array was analysed using Image Lab (Bio-Rad) and GIMP.

Quantitative polymerase chain reaction (qPCR)

Cells (100,000/well) were seeded into 6 well plates. After 24 hours, media was replenished with fresh media without TKI addition. After a further 72 hours, cells were lysed and RNA extracted using QIAshredder and RNeasy kits (Qiagen), following the manufacturer's recommendations. Contaminating DNA was degraded by RQ1 RNase-free DNase (Promega) followed by cDNA synthesis utilising the SuperScript III First-Strand Synthesis kit (ThermoFisher Scientific), following the manufacturer's recommendations. qPCR was undertaken using SYBR green fluorescent dye (ThermoFisher Scientific), following the manufacturer's recommendations, on Applied Biosystems QuantStudio 6 or 7 Flex Real-Time PCR systems (ThermoFisher Scientific). PCR conditions were as follows: 120 secs 50 ºC (hold), 120 secs 95 ºC (hold), 15 secs 95ºC (40 cycles), and 60 secs 60ºC (40 cycles).

Primers (Sigma Aldrich) used were for ACTB (forward: GACAGGATGCAGAAGGAGATCAC and reverse: TGATCCACATCTGCTGGAAGGT), PDGFRA (forward: GACTTTCGCCAAAGTGGAGGAG and reverse: AGCCACCGTGAGTTCAGAACGC),

FGFR1 (forward: GCACATCCAGTGGCTAAAGCAC and reverse: AGCACCTCCATCTCTTTGTCGG), FGFR3 (forward: TCCATCTCCTGGCTGAAGAACG and reverse: TGTTCTCCACGACGCAGGTGTA) and FGFR4 (forward: AACACCGTCAAGTTCCGCTGTC and reverse: CATCACGAGACTCCAGTGCTGA). A melt curve was undertaken post-reaction to assess primer and experimental quality.

FGFR shRNA knockdown

The control shRNA (shCont) and shRNA sequences for *FGFR1, FGFR3 and FGFR4* were obtained from the RNAi consortium of the Broad Institute (sequences provided in Short hairpin sequences table). Oligos (Sigma-Aldrich) were cloned into the lentiviral Tet-pLKO-puro vector (Addgene #21915). Plasmid sequences were confirmed using Sanger sequencing.

Short hairpin sequences

shRNA sequences used for cloning and knockdown of FGFR1, FGFR3 and FGFR4 in A204 and G402 sitravatinib resistant sublines.

	Forward (5'-3')	Reverse (5'-3')
shControl	CCGGCAACAAGATGAAGAGCACCAACTCGAGTTGGTGCTCTTCATCTTGTTGTTTTTG	AATTCAAAAACAACAAGATGAAGAGCACCAACTCGAGTTGGTGCTCTTCATCTTGTTG
FGFR1 shRNA 1	CCGGGCCAAGACAGTGAAGTTCAAACTCGAGTTTGAACTTCACTGTCTTGGCTTTTTG	AATTCAAAAAGCCAAGACAGTGAAGTTCAAACTCGAGTTTGAACTTCACTGTCTTGGC
FGFR1 shRNA 2	CCGGGAATGAGTACGGCAGCATCAACTCGAGTTGATGCTGCCGTACTCATTCTTTTTG	AATTCAAAAAGAATGAGTACGGCAGCATCAACTCGAGTTGATGCTGCCGTACTCATTC
FGFR3 shRNA 1	CCGGGTGATTCCAGTGAAGATATTTCTCGAGAAATATCTTCACTGGAATCACTTTTTG	AATTCAAAAAGTGATTCCAGTGAAGATATTTCTCGAGAAATATCTTCACTGGAATCAC
FGFR3 shRNA 2	CCGGGACAAGGAGCTAGAGGTTCTCCTCGAGGAGAACCTCTAGCTCCTTGTCTTTTTG	AATTCAAAAAGACAAGGAGCTAGAGGTTCTCCTCGAGGAGAACCTCTAGCTCCTTGTC
FGFR4 shRNA 1	CCGGATCTACCTCTCGACCCACTATCTCGAGATAGTGGGTCGAGAGGTAGATTTTTTG	AATTCAAAAAATCTACCTCTCGACCCACTATCTCGAGATAGTGGGTCGAGAGGTAGAT
FGFR4 shRNA 2	CCGGAGACATCAATAGCTCAGAGGTCTCGAGACCTCTGAGCTATTGATGTCTTTTTTG	AATTCAAAAAAGACATCAATAGCTCAGAGGTCTCGAGACCTCTGAGCTATTGATGTCT

PDGFRA expression experiments

The empty vector plasmid pHAGE plasmid (#126686) and pHAGE-PDGFRA-Y288C were purchased from Addgene (#116453)[4]. Site-directed mutagenesis (New England Biolabs Q5 kit) was used to revert the PDGFRA-Y288C to the wild type PDGFRA sequence and engineer the T674M mutant. Primers used were designed using NEBaseChanger. Sanger sequencing was used to confirm plasmid sequence.

Lentiviral transduction

Lentivirus was produced by transfecting HEK-293T cells with 3.3 μg insert DNA, 1.5 μg pMD2.G (Addgene #12259) and 3.3 μg psPAX2 (Addgene #12260). Viral media was collected, filtered through 0.45 μm pore filter and used to infect A204SitR cells with 8 μg/ml polybrene (Sigma-Aldrich) for 24 h. Cells infected with constructs containing a puromycin selection gene were selected using 2 μg/ml puromycin (Sigma-Aldrich).

RNA sequencing and analysis

A204 and G402 were seeded at 100,000 cells/6 well were seeded, media was changed after 24 hours and cells trypsinsed and cell pellets collected 96 hours after seeding. RNA was collected using RNeasy kit (Qiagen). mRNA library preparation with PolyA enrichment and 150 million paired end reads using NovaSeq 6000 was carried out by Novogene, along with alignment adaptor trimming and calculation of fragments per kilobase of transcript per million mapped reads (FPKM). FPKM values were converted to transcripts per million (TPM) by dividing by total FPKM and multiplying by one million. Significance analysis of microarrays (SAM) analysis was performed using the *samr* (v3.0; https://cran.r-project.org/web/packages/samr/index.html) package to identify differentially expressed genes. Two-class unpaired SAM analysis was used to compare drug-resistant cell lines versus parental cell lines. A204 and G402 cell lines were combined for this analysis. Volcano plot visualisations were created using the *ggplot2* (v3.5.2; https://ggplot2.tidyverse.org/) package. Gene set enrichment analysis was performed using the *fgsea* (v3.21;

https://bioconductor.org/packages/release/bioc/html/fgsea.html) package to identify enriched pathways from the Gene Ontology: Biological Pathways list. Multiple testing correction was applied using the Benjamini-Hochberg method[5], and a false discovery rate of 1% was considered the threshold for significance. To identify uniquely differentially expressed genes, two-class unpaired SAM analysis was used to compare cells resistant to each drug against the others (e.g. anlotinib resistant versus all others). Visualisation was performed using the *ComplexHeatmap* package (v3.21; https://bioconductor.org/packages/release/bioc/html/ComplexHeatmap.html). All analyses were performed in R v4.4.0.

Cell lines

Details of cell lines used in this study

Cell line	Subtype	Cellosaurus accession code[6]
A204	MRT	CVCL_1058
G402	MRT	CVCL_1221
HT1080	Fibrosarcoma	CVCL_0317
SW684	Fibrosarcoma	CVCL_1726
MESSA	Uterine sarcoma	CVCL_1404
SW872	Liposarcoma	CVCL_1730
Hs729T	eRMS	CVCL_0871
RMS-YM	eRMS	CVCL_A792
RUCH3	eRMS	CVCL_C541
SW982	Synovial sarcoma	CVCL_1734
SAOS2	Osteosarcoma	CVCL_0548
U2OS	Osteosarcoma	CVCL_0042
SJSA1	Osteosarcoma	CVCL_1697
T91-95	aRMS	n/a [7,8]

aRMS; Alveolar rhabdomysarcoma, CVCL; Cellosaurus accession code, eRMS; embryonal rhabdomyosarcoma, MRT; Malignant rhabdoid tumour.

Small molecule inhibitors

Details of small molecule inhibitors used in this study

Small molecule inhibitor	Primary target(s)	Supplier
Cediranib	Broad spectrum: RTKs	LC Laboratories
Foretinib	Broad spectrum: RTKs	LC Laboratories
Imatinib	Broad spectrum: RTKs, Abl1	LC Laboratories
Lenvatinib	Broad spectrum: RTKs	LC Laboratories
Pazopanib	Broad spectrum: RTKs	LC Laboratories
Ponatinib	Broad spectrum: RTKs, Abl1	LC Laboratories
Regorafenib	Broad spectrum: RTKs	LC Laboratories
Sitravatinib	Broad spectrum: RTKs	Selleck Chemicals
Sorafenib	Broad spectrum: RTKs, C-Raf, B-Raf	LC Laboratories
Sunitinib	Broad spectrum: RTKs	LC Laboratories
Vandetanib	Broad spectrum: RTKs	LC Laboratories
Entrectinib	NTRK1/2/3, ROS1, ALK	Selleck Chemicals
GW441756	NTRK1	Selleck Chemicals
Ceritinib	ALK	Selleck Chemicals
Crizotinib	ALK, MET	LC Laboratories
NVP-TAE684	ALK	Selleck Chemicals
Osimertinib (AZD-9291)	EGFR	Selleck Chemicals
EAI045	EGFR	Selleck Chemicals
Erlotinib	EGFR	LC Laboratories
Gefitinib	EGFR	LC Laboratories
Lapatinib	EGFR, HER2	LC Laboratories
Neratinib	EGFR, HER2	LC Laboratories
Infigratinib (BGJ-398)	FGFR1/2/3	Selleck Chemicals
Linsitinib	IGF1R	LC Laboratories
NVP-AEW541	IGF1R, InsR	Selleck Chemicals
Cilengitide trifluoroacetate	Integrins αvβ3, αvβ5	Selleck Chemicals
Bosutinib	Src, Abl1	LC Laboratories
Dasatinib	Src, Abl1, Broad spectrum: RTKs	LC Laboratories
Saracatinib	Src	Selleck Chemicals
PF562271	FAK	Selleck Chemicals
TAE226	FAK	Selleck Chemicals
BI-2536	PLK1	Selleck Chemicals
BX-795	PDPK1	Sigma Aldrich
Dactolisib (BEZ235)	PI3K, mTOR	LC Laboratories
Rapamycin (Sirolimus)	mTOR	LC Laboratories
Binimetinib	MEK1/2	LC Laboratories
Trametinib	MEK1/2	LC Laboratories
Dabrafenib	B-Raf V600E	Selleck Chemicals
Adezmapimod (SB203580)	p38 MAPK	Selleck Chemicals
SP600125	JNK1/2/3	Selleck Chemicals
Capivasertib (AZD-5363)	Akt1/2/3	Selleck Chemicals
MK2206	Akt1/2/3	Selleck Chemicals
Momelotinib	JAK1/2	Selleck Chemicals
Niclosamide	STAT3	Selleck Chemicals
SH-4-54	STATs	Selleck Chemicals
Galunisertib	TGFβR1	Selleck Chemicals
BMS345541	IKK1/2	Sigma Aldrich
Alisertib	Aurora A	Selleck Chemicals
Rabusertib (LY2603618)	Chk1	Selleck Chemicals
MK8776	Chk1	Selleck Chemicals
Palbociclib	CDK4/6	Selleck Chemicals
Silmitasertib	CK2	Selleck Chemicals
Talazoparib	PARP	Selleck Chemicals
Rucaparib	PARP	Selleck Chemicals
XAV-939	Tankyrase	Selleck Chemicals
Navitoclax	Bcl-2, Bcl-w, Bcl-xL	Selleck Chemicals
GSK126	EZH2	Selleck Chemicals
JQ1	BET bromodomains	Selleck Chemicals
Luminespib (NVP-AUY922)	Hsp90	LC Laboratories
ADP; Adenosine diphosphate, ALK; Anaplastic lymphoma kinase, Bcl-(2/xL); B-cell lymphoma (2/extra large) protein; Bcl-w; Bcl-2-like protein 2, Bcr; Breakpoint cluster region protein, BET; Bromo- and extra-terminal domain, CDK(4/6); Cyclin-dependent kinase (4/6); Chk1; Checkpoint kinase 1, CK2; Casein kinase 2, EGFR; Epidermal growth factor, EZH2; Enhancer of zeste homolog 2, FAK; Focal adhesion kinase, FGFR(1/2/3/4); Fibroblast growth factor receptor (1/2/3/4), HER2; Human epidermal growth factor		

receptor 2, Hsp90; Heat shock protein 90, IGF1R; Insulin-like growth factor 1 receptor, IKK(1/2); IκB kinase (1/2), InsR; Insulin receptor, JAK(1/2); Janus kinase (1/2), JNK(1/2/3); c-Jun N-terminal kinase (1/2/3), MAPK; Mitogen-activated protein kinase, MEK; Mitogen-activated protein kinase kinase, mTOR; Mechanistic target of rapamycin, N-terminal; Amino-terminal, NTRK(1/2/3); Neurotrophic tyrosine kinase receptor (1/2/3) PARP; Poly (ADP-ribose) polymerase, PDPK1; Phosphoinositide-dependent protein kinase 1, PI3K; Phosphoinositide 3-kinase, PLK1; Polo-like kinase 1, (B/C)-Raf; Rapidly accelerated fibrosarcoma, RTK; Receptor tyrosine kinase, STAT(3); Signal transducer and activator of transcription (3), TGFβR1; Transforming growth factor β receptor 1.

References

1. Wisniewski, J.R., Zougman, A., Nagaraj, N. & Mann, M. Universal sample preparation method for proteome analysis. *Nat Methods* **6**, 359-362 (2009).
2. Hastie, T., Tibshirani, R., Narasimhan, B. & Chu, G. impute: impute:Imputation for microarray data. R package version 1.64.0. (2020).
3. Gu, Z., Eils, R. & Schlesner, M. Complex heatmaps reveal patterns and correlations in multidimensional genomic data. *Bioinformatics* **32**, 2847-2849 (2016).
4. Ng, P.K.*, et al.* Systematic Functional Annotation of Somatic Mutations in Cancer. *Cancer Cell* **33**, 450-462 e410 (2018).
5. Benjamini, Y. & Hochberg, Y. Controlling the False Discovery Rate - a Practical and Powerful Approach to Multiple Testing. *J R Stat Soc B* **57**, 289-300 (1995).
6. Bairoch, A. The Cellosaurus, a Cell-Line Knowledge Resource. *J Biomol Tech* **29**, 25-38 (2018).
7. Lee, Y.F.*, et al.* Molecular classification of synovial sarcomas, leiomyosarcomas and malignant fibrous histiocytomas by gene expression profiling. *Br J Cancer* **88**, 510-515 (2003).
8. Martins, A.S., Olmos, D., Missiaglia, E. & Shipley, J. Targeting the insulin-like growth factor pathway in rhabdomyosarcomas: rationale and future perspectives. *Sarcoma* **2011**, 209736 (2011).
