# Supplementary material for: Exploiting collateral sensitivity in the evolution of resistance to tyrosine kinase inhibitors in soft tissue sarcomas
Source: Commun Biol. 2025 Aug 8;8:1185. doi: 10.1038/s42003-025-08652-1 (PMC12334625; doi:10.1038/s42003-025-08652-1)
Supplement: Supplementary file 3 — Description of Additional Supplementary Files [file 42003_2025_8652_MOESM3_ESM.pdf]

**Description of Additional Supplementary Files**

File name- Supplementary Data

File description – All source data is for graphs.
