# Supplementary material for: Exploiting collateral sensitivity in the evolution of resistance to tyrosine kinase inhibitors in soft tissue sarcomas
Source: Commun Biol. 2025 Aug 8;8:1185. doi: 10.1038/s42003-025-08652-1 (PMC12334625; doi:10.1038/s42003-025-08652-1)
Supplement: Supplementary file 4 — Reporting Summary [file 42003_2025_8652_MOESM4_ESM.pdf]

nature portfolio

Corresponding author(s): Huang

Last updated by author(s): Jul 29, 2025

Reporting Summary

Nature Portfolio wishes to improve the reproducibility of the work that we publish. This form provides structure for consistency and transparency in reporting. For further information on Nature Portfolio policies, see our Editorial Policies and the Editorial Policy Checklist.

Statistics

For all statistical analyses, confirm that the following items are present in the figure legend, table legend, main text, or Methods section.

n/a	Confirmed	
☐	☒	The exact sample size (*n*) for each experimental group/condition, given as a discrete number and unit of measurement
☐	☒	A statement on whether measurements were taken from distinct samples or whether the same sample was measured repeatedly
☐	☒	The statistical test(s) used AND whether they are one- or two-sided<br>*Only common tests should be described solely by name; describe more complex techniques in the Methods section.*
☐	☒	A description of all covariates tested
☐	☒	A description of any assumptions or corrections, such as tests of normality and adjustment for multiple comparisons
☐	☒	A full description of the statistical parameters including central tendency (e.g. means) or other basic estimates (e.g. regression coefficient) AND variation (e.g. standard deviation) or associated estimates of uncertainty (e.g. confidence intervals)
☐	☒	For null hypothesis testing, the test statistic (e.g. *F*, *t*, *r*) with confidence intervals, effect sizes, degrees of freedom and *P* value noted<br>*Give P values as exact values whenever suitable.*
☒	☐	For Bayesian analysis, information on the choice of priors and Markov chain Monte Carlo settings
☒	☐	For hierarchical and complex designs, identification of the appropriate level for tests and full reporting of outcomes
☒	☐	Estimates of effect sizes (e.g. Cohen's *d*, Pearson's *r*), indicating how they were calculated

Our web collection on statistics for biologists contains articles on many of the points above.

Software and code

Policy information about availability of computer code

Data collection	mRNA library preparation with PolyA enrichment and 150 million paired end reads using NovaSeq 6000 was carried out by Novogene, along with alignment adaptor trimming and calculation of fragments per kilobase of transcript per million mapped reads (FPKM).
Data analysis	RNA sequence data was performed using the samr (v3.0; https://cran.r-project.org/web/packages/samr/index.html) package to identify differentially expressed genes. Volcano plot visualisations were created using the ggplot2 (v3.5.2; https://ggplot2.tidyverse.org/) package. Gene set enrichment analysis was performed using the fgsea (v3.21; https://bioconductor.org/packages/release/bioc/html/fgsea.html). Visualisation was performed using the ComplexHeatmap package (v3.21; https://bioconductor.org/packages/release/bioc/html/ComplexHeatmap.html). All analyses were performed in R v4.4.0.

For manuscripts utilizing custom algorithms or software that are central to the research but not yet described in published literature, software must be made available to editors and reviewers. We strongly encourage code deposition in a community repository (e.g. GitHub). See the Nature Portfolio guidelines for submitting code & software for further information.

Data

Policy information about availability of data

All manuscripts must include a data availability statement. This statement should provide the following information, where applicable:

- Accession codes, unique identifiers, or web links for publicly available datasets
- A description of any restrictions on data availability
- For clinical datasets or third party data, please ensure that the statement adheres to our policy

Raw RNAseq data have been deposited to GEO (series record GSE298261). All other data supporting the findings of this study are available from the corresponding author (PHH) upon reasonable academic request and will require the researcher to sign a data access agreement with the Institute of Cancer Research after approval.

Research involving human participants, their data, or biological material

Policy information about studies with human participants or human data. See also policy information about sex, gender (identity/presentation), and sexual orientation and race, ethnicity and racism.

Reporting on sex and gender	No human participant data was used in this study.
Reporting on race, ethnicity, or other socially relevant groupings	No human participant data was used in this study.
Population characteristics	No human participant data was used in this study.
Recruitment	No human participant data was used in this study.
Ethics oversight	No human participant data was used in this study.

Note that full information on the approval of the study protocol must also be provided in the manuscript.

Field-specific reporting

Please select the one below that is the best fit for your research. If you are not sure, read the appropriate sections before making your selection.

☒ Life sciences ☐ Behavioural & social sciences ☐ Ecological, evolutionary & environmental sciences

For a reference copy of the document with all sections, see nature.com/documents/nr-reporting-summary-flat.pdf

Life sciences study design

All studies must disclose on these points even when the disclosure is negative.

Sample size	No statistical method was used to determine sample size. Our sample sizes are similar to those reported in previous publications using similar experiments.
Data exclusions	No data was excluded from the analysis.
Replication	Number of replicates is reported in legends, unless stated otherwise all experiments were tested in a minimum of 3 independent experiments.
Randomization	Randomization was not used in this study as all experiments were cell line based. Due to the lack of experimental variability of these models this eliminated the need for randomization.
Blinding	No blinding was used in this study as the cell line based assays do not introduce bias as would occur in a participant based clinical trial.

Reporting for specific materials, systems and methods

We require information from authors about some types of materials, experimental systems and methods used in many studies. Here, indicate whether each material, system or method listed is relevant to your study. If you are not sure if a list item applies to your research, read the appropriate section before selecting a response.

Materials & experimental systems

n/a	Involved in the study
☐	☒ Antibodies
☐	☒ Eukaryotic cell lines
☒	☐ Palaeontology and archaeology
☒	☐ Animals and other organisms
☒	☐ Clinical data
☒	☐ Dual use research of concern
☒	☐ Plants

Methods

n/a	Involved in the study
☒	☐ ChIP-seq
☒	☐ Flow cytometry
☒	☐ MRI-based neuroimaging

Antibodies

Antibodies used

rabbit anti-pAkt (S473) (1:1000; 193H12; Cell Signalling Technology; 4058)
rabbit anti-Akt (pan) (1:1000; C67E7, Cell Signalling Technology; 4691)
rabbit anti-p44/42 MAPK (ERK1/2) (T202/Y204) (1:1000; D13.14.4E; Cell Signalling Technology; 4370)
rabbit anti-p44/42 MAPK (ERK1/2) (1:1000; 137F5; Cell Signalling Technology; 4695)
rabbit anti-PDGFRA (1:1000; D1E1E; Cell Signalling Technology; 3174)
rabbit Anti-FGFR1 (1:1000; EPR806Y; Abcam; ab76464)
mouse anti-α-tubulin (1:5000; Sigma Aldrich; T5168)
rabbit anti-pPDGFRA (Y754) (1:500; 23B2; Cell Signalling Technology; 2992)
rabbit anti-pPDGFRA (Y1018) (1:500; Cell Signalling Technology; 4547)

Validation

Details for validation are provided in the following manufacturer links.
anti-pAkt (S473) https://www.cellsignal.com/products/primary-antibodies/phospho-akt-ser473-193h12-rabbit-mab/4058
anti-Akt (pan) https://www.cellsignal.com/products/primary-antibodies/akt-pan-c67e7-rabbit-mab/4691
anti-p44/42 MAPK (ERK1/2) (T202/Y204) https://www.cellsignal.com/products/primary-antibodies/phospho-p44-42-mapk-erk1-2-thr202-tyr204-d13-14-4e-xp-rabbit-mab/4370
anti-p44/42 MAPK (ERK1/2) https://www.cellsignal.com/products/primary-antibodies/p44-42-mapk-erk1-2-137f5-rabbit-mab/4695?_=1678268526254&Ntt=4695&tahead=true
anti-PDGFRA https://www.cellsignal.com/products/primary-antibodies/pdgf-receptor-a-d1e1e-xp-rabbit-mab/3174
Anti-FGFR1 https://www.abcam.com/products/primary-antibodies/fgfr1-antibody-epr806y-ab76464.html
anti-α-tubulin https://www.sigmaaldrich.com/GB/en/product/sigma/t5168
anti-pPDGFRA (Y754) https://www.cellsignal.com/products/primary-antibodies/phospho-pdgf-receptor-a-tyr754-23b2-rabbit-mab/2992
anti-pPDGFRA (Y1018) https://www.cellsignal.com/products/primary-antibodies/phospho-pdgf-receptor-a-tyr1018-antibody/4547

Eukaryotic cell lines

Policy information about cell lines and Sex and Gender in Research

Cell line source(s)

A204 (https://www.atcc.org/products/htb-82#documentation) and G402 (https://www.atcc.org/products/crl-1440) purchased from ATCC.
From Janet Shipley (Divisions of Molecular Pathology and Cancer Therapeutics, The Institute of Cancer Research) SAOS2, U2OS, HT1080, MESSA, SW684, SW872, Hs729T, RUCH3, T91-95, SW982, SJSA1 and RMS-YM.

Authentication

All cell lines were authenticated.

Mycoplasma contamination

Cell lines mycoplasma free, tested using InvivoGen Mycoplasma strip test (Cat no. rep-mys-100).

Commonly misidentified lines
(See ICLAC register)

No commonly misidentified lines were used in this study.

Plants

Seed stocks

Not relevant as this study did not use plants.

Novel plant genotypes

Not relevant as this study did not use plants.

Authentication

Not relevant as this study did not use plants.
